# Genomic Resequencing Unravels the Genetic Basis of Domestication, Expansion, and Trait Improvement in *Morus Atropurpurea*

**DOI:** 10.1002/advs.202300039

**Published:** 2023-06-20

**Authors:** Fanwei Dai, Xiaokang Zhuo, Guoqing Luo, Zhenjiang Wang, Yujuan Xu, Dan Wang, Jianwu Zhong, Sen Lin, Lian Chen, Zhiyi Li, Yuan Wang, Diyang Zhang, Yuanyuan Li, Qinyao Zheng, Tangchun Zheng, Zhong‐Jian Liu, Li Wang, Zhiyong Zhang, Cuiming Tang

**Affiliations:** ^1^ Sericultural & Agri‐Food Research Institute Guangdong Academy of Agricultural Sciences/Key Laboratory of Functional Foods Ministry of Agriculture and Rural Affairs/Guangdong Key Laboratory of Agricultural Products Processing Guangzhou 510610 P. R. China; ^2^ Key Laboratory of Urban Agriculture in South China Ministry of Agriculture and Rural Affairs Guangzhou 510610 P. R. China; ^3^ College of Horticulture Fujian Agriculture and Forestry University Fuzhou 350002 P. R. China; ^4^ National Engineering Research Center for Floriculture Beijing Forestry University Beijing 100083 P. R. China; ^5^ Key Laboratory of National Forestry and Grassland Administration for Orchid Conservation and Utilization at College of Landscape Architecture Fujian Agriculture and Forestry University Fuzhou 350002 P. R. China; ^6^ Shenzhen Branch Guangdong Laboratory of Lingnan Modern Agriculture Genome Analysis Laboratory of the Ministry of Agriculture and Rural Affairs Agricultural Genomics Institute at Shenzhen Chinese Academy of Agricultural Sciences Shenzhen 518120 P. R. China; ^7^ Kunpeng Institute of Modern Agriculture at Foshan Chinese Academy of Agricultural Sciences Foshan 528225 P. R. China; ^8^ Beijing Advanced Innovation Center for Tree Breeding by Molecular Design Beijing University of Agriculture Beijing 102206 P. R. China

**Keywords:** domestication, evolutionary history, flowering time, genome, mulberry

## Abstract

Mulberry is an economically important plant in the sericulture industry and traditional medicine. However, the genetic and evolutionary history of mulberry remains largely unknown. Here, this work presents the chromosome‐level genome assembly of *Morus atropurpurea* (*M. atropurpurea*), originating from south China. Population genomic analysis using 425 mulberry accessions reveal that cultivated mulberry is classified into two species, *M. atropurpurea* and *M. alba*, which may have originated from two different mulberry progenitors and have independent and parallel domestication in north and south China, respectively. Extensive gene flow is revealed between different mulberry populations, contributing to genetic diversity in modern hybrid cultivars. This work also identifies the genetic architecture of the flowering time and leaf size. In addition, the genomic structure and evolution of sex‐determining regions are identified. This study significantly advances the understanding of the genetic basis and domestication history of mulberry in the north and south, and provides valuable molecular markers of desirable traits for mulberry breeding.

## Graphical abstract

Demographic history of mulberry revealed that *M. atropurpurea* and *M. alba* might have originated from two different mulberry progenitors and had independent and parallel domestication in north and south China, respectively. Extensive gene flow is revealed between different mulberry populations, contributing to genetic diversity in modern hybrid cultivars.

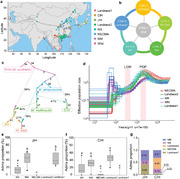

## 1 Introduction

Plant domestication is an artificial selection process that genetically and morphologically modifies wild‐type progenitors for human use.^[^ ^1^ ^]^ Over 2000 plant species have been domesticated worldwide.^[^ ^2^ ^]^ To date, studies of plant domestication, improvement, and expansion have largely focused on economically important or highly domesticated annual crops or vbles, such as soybean,^[^ ^3^, ^4^ ^]^ maize,^[^ ^5^ ^]^ wheat,^[^ ^6^ ^]^ rice,^[^ ^1^, ^7^ ^]^ sorghum,^[^ ^8^ ^]^ tomato,^[^ ^9^ ^]^ and cucumber.^[^ ^10^ ^]^ Insights into the domestication of woody fruit plants have also been previously reported. For instance, a genome‐wide association study showed that two candidate genes were highly associated with fruit shape and non‐acidity in peach,^[^ ^11^ ^]^ and several wild crabapples have contributed to the genome of the domesticated apple.^[^ ^12^ ^]^ Evidence has demonstrated that apple fruit size experienced two rounds of enlargement occurring before and during domestication.^[^ ^13^ ^]^ Studies on perennial domestication and trait evolution will not only improve its insufficient representation but also provide valuable and novel insights into the genetics of plant domestication.

Mulberry (*Morus* spp.) is a well‐known economic plant that is widely cultivated in most developing countries in Asia. As the only food source for silkworms (*Bombyx mori* L.), mulberry is a vital component in the development of the sericulture industry, ancient Silk Road, and cultural exchanges.^[^ ^14^ ^]^ Mulberry was reported to originate in China and has a cultivation history of more than 5000 years.^[^ ^15^ ^]^ The domestication of mulberry increased leaf size and yield, altered flowering time, and improved its adaptivity to variable environments across latitudinal gradients.^[^ ^16^, ^17^, ^18^ ^]^ However, little is known about the genetic basis and domestication history of these key agronomic and economic traits of mulberry plants.

Mulberry trees are widely planted in Eurasia, Africa, Oceania, and America, and more than 1000 domesticated mulberry germplasms are grown in a wide range of agroclimatic regions in China.^[^ ^19^ ^]^ Mulberry diversity is the highest in China, with 24 of the 68 species in the world.^[^ ^17^ ^]^ According to the traditional taxonomic system, the cultivars used in sericulture belong to five species: *Morus alba* (*M. alba*), *Morus multicaulis* (*M. multicaulis*), *Morus bombycis* (*M. bombycis*), *Morus atropurpurea* (*M. atropurpurea*), and *Morus mizuho* (*M. mizuho*).^[^ ^14^ ^]^ However, three species (*M. alba*, *M. multicaulis*, and *M. atropurpurea*) are widely cultivated for their leaves to feed silkworms.^[^ ^14^ ^]^ In China, ≈70% of the approved mulberry varieties belong to *M. alba*, *M. multicaulis*, or *M. atropurpurea*, and the rest are hybrids (crosses between *M. atropurpurea* and *M. alba* or *M. multicaulis*).^[^ ^14^ ^]^ These highly domesticated species have spread to multiple areas, including India, Europe, and America.^[^ ^20^ ^]^ Recently, researchers reported the chromosome‐level genome of *M. alba* planted in north China and revealed its genetic basis for environmental adaptation based on whole‐genome resequencing data of 134 accessions of *M. alba*.^[^ ^16^ ^]^ However, this genome does not represent the genetic diversity of a key subtropical representative of mulberry, *M. atropurpurea*, and thus hinders our understanding of the domestication history of mulberries and their expansion route into other regions.

This study aimed to gain an in‐depth understanding of the long‐term domestication and geographical expansion of mulberry and to dissect the genomic basis controlling major agronomic traits. We first reported chromosome‐scale genomes of *M. atropurpurea* selected from two elite cultivars, a male cultivar “Huiqiu1” and a female cultivar “Tang10” which widely planted in southern China. Based on the assembled genome, we assessed the genetic diversity among geographically distinct populations of 425 individuals (290 accessions were sequenced in this study, and 135 accessions were previously published.^[^ ^16^, ^21^ ^]^ Our study provides insights into the domestication history and genetic architecture of key agronomic traits in mulberry, and expands our knowledge of perennial domestication.

## 2 Results

### 2.1 De Novo Assembly and Comparative Genomics of M. Atropurpurea

**Figure 1:**
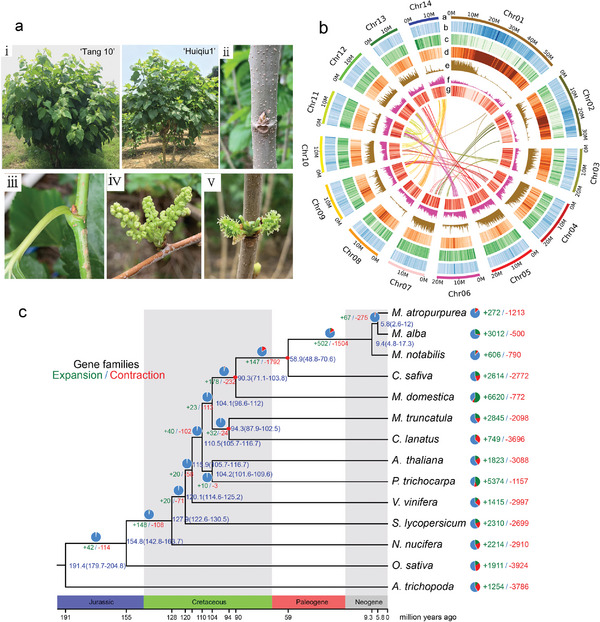
*De novo* assembly of the *M. atropurpurea* ‘Huiqiu1’genome and gene family expansions and contractions throughout evolution. a) The plant of *M. atropurpurea* was used for genome assembly in this study. i) tree; ii) dormant bud; iii) axillary bud; iv) male flower; v) female flower. b) Circos plot showing the landscape of the *M. atropurpurea* genome. Outermost to innermost tracks indicate the a) pseudochromosomes, b) GC content, c) gene density, d) repeat density, e) gene expression, f) pseudogene density , and g) single nucleotide polymorphism (SNP) density. c) Species tree with molecular dating and gene family expansion and contraction indicated. The phylogenetic tree was constructed, and the divergence time (in MYA) was estimated based on single‐copy genes from 12 gene families. Gene family expansions and contractions on different lineages and species tips are indicated with green and red colors, respectively. The red dot represents the correction of time point using the speciation time of *A. thaliana* and *P. trichocarpa* (102.0–113.8 MYA), *M. truncatula* and *C. lanatus* (89.2–104.5 MYA), *M. domestica* and *C. sativa* (73.6–90.2 MYA) based on TIMATREE5 (http://timetree.org/).

Based on the analysis of chromosome ploidy (Figure S1, Supporting Information), a diploid (2*n* = 28) *M. atropurpurea* male cultivar “Huiqiu1” (Figure 1) was selected for whole‐genome sequencing using Illumina HiSeq, PacBio, and high‐throughput chromatin conformation capture (Hi‐C) (Figure S2, Supporting Information). After filtering out low‐quality reads, a total of 36.8 Gb PacBio long reads (119× assuming a genome size of 0.308 Gb estimated by flow cytometry), 9.2 Gb Illumina paired‐end reads (30×), and 30.3 Gb Hi‐C data (98×) were obtained. The genome of “Huiqiu1” was assembled into two haplotypes. The haplotype A was 305.25 Mb, consisting of 45 contigs, with an N50 of 16.35 Mb, while haplotype B was 310.49 Mb, consisting of 40 contigs, with an N50 of 14.73 Mb (Table 1). The genome size was consistent with estimates from flow cytometry and genome surveys (Figure S3, Supporting Information). Contigs of male cultivar “Huiqiu1” were then corrected and scaffolded, about of 99% of which were anchored to 14 pseudochromosomes (Figure 1b and Table S1, Supporting Information). Finally, 305.25 and 310.49 Mb assembled genome of two haplotypes were obtained from male cultivar “Huiqiu1” with scaffold N50 of 20.47 and 21.53 Mb, respectively (Table 1). Meanwhile, a diploid *M. atropurpurea* female cultivar “Tang10” was also selected for whole‐genome sequencing using Illumina HiSeq, Oxford Nanopore, and high‐ Hi‐C. After filtering out low‐quality reads, a total of 100.1 Gb Oxford Nanopore long reads (304×assuming a genome size of 0.308 Gb estimated by flow‐cytometry), 38.5 Gb Illumina paired‐end reads (117×), and 42.1 Gb Hi‐C data (128×) were obtained. The assembled genome was 328.97 Mb, consisted of 264 contigs with an N50 of 2.44 Mb (Table 1). Contigs were then corrected and scaffolded by Hi‐C, 98.75% of which could be anchored to 14 pseudochromosomes. Finally, 328.97 Mb assembled genome with a scaffold N50 of 21.53 Mb was obtained from female cultivar “Tang10” (Table 1).

**Table 1:** Comparisons of the assemblies and annotations of de novo assembled *M. atropurpurea* genome with published *M. alba* and *M. notabilis* genomes

Parameter	*M. atropurpurea* (Male)		*M. atropurpurea* (Female)	*M. alba*	*M. notabilis*
	Haplotype A	Haplotype B			
Genome size [Mb]	305.25	310.49	328.97	346.39	301.54
No. of contigs	45	40	264	398	539
N50 of contigs [Mb]	16.35	14.73	2.44	2.71	2.71
No. of scaffolds	28	23	54	16	542 681
N50 of scaffolds [Mb]	20.47	21.06	21.53	22.87	0.3955.32
No. of unanchored scaffolds	11	12	40	236	536
Unanchored length [Mb]	1.38	1.48	7.75	10.34	10.64
GC content [%]	34.72	34.81	34.67	34.29	35.11
No. of genes	21 092	20 901	25 675	22 767	25 391
Average gene length [bp]	4524	4548	4159	3209	3140
Average CDS length [bp]	1357	1358	1230	1148	164
Average exon number per gene	6.6	6.6	5.4	5.09	86
Repeat sequence length [Mb]	163.85	168.65	173.15	180.11	164.41
Percentage of repeat sequences [%]	53.68	54.43	52.63	52.85	54.52
Chromosome number	28	28	28	28	12
Chromosome length [%]	99.90	99.18	98.88	98.38	96.4
Complete BUSCOs [%]	97.4	97.6	96.47	94.30	93.60
Reference	This study	This study	This study	[16]	[18]

Collinearity analysis showed that the assembled genome of *M. atropurpurea* matched well with *M. alba* ^[^ ^16^ ^]^ (Figure S4, Supporting Information). Comparative analysis of the genomes of the two species showed that chromosome 1 of *M. alba* was ≈8–12 Mb longer than that of *M. atropurpurea* (Table S1, Supporting Information), and 343 365 highly divergent regions (HDRs) were identified (Figure S5 and Table S2, Supporting Information). About 92% (231) of highly conserved eukaryotic core genes (CEGs) were identified using the Core Eukaryotic Genes Mapping Approach (CEGMA) analysis, and about 97% of the complete CEGs were present in the *M. atropurpurea* genome based on Benchmarking Universal Single‐Copy Orthologs (BUSCO) analysis (Table 1 and Table S3, Supporting Information). The extensive coverage of core plant genes, together with the high collinearity with *M. alba*, indicated that the assembly was highly complete and accurate. We identified 21 092 and 20 901 protein‐coding genes from the haplotype A and haplotype B of male genome, respectively; while 25 675 protein‐coding genes were identified from the female genome (Table 1). Among them, more than 98% genes were functionally annotated (Table S4, Supporting Information). About 50% of the genome was composed of repetitive sequences (Tables S5 and S6, Supporting Information), and about 700 non‐coding RNAs (rRNA, tRNA, and miRNA) were predicted (Table S7, Supporting Information).

The phylogenetic and molecular dating analyses showed that *M. atropurpurea* and *M. alba* were diversified from their relative *M. notabilis* ≈14.8 million years ago (MYA), which was similar to that of the previous study (≈5.5–17.1 MYA) between *M. alba* and *M. notabilis*.^[^ ^16^ ^]^ The speciation time between *M. atropurpurea* and *M. alba* was ≈5.8 MYA. *M. atropurpurea* split from its nearest family, Cannabaceae, represented by *Cannabis sativa*, at ≈58.9 MYA (Figure 1c), which is consistent with the time of the reported mulberry leaf fossil.^[^ ^24^ ^]^ Genomic synteny analysis showed a 1:2 syntenic relationship between *M. atropurpurea* and *P. trichocarpa* and a 1:1 syntenic relationship between *M. atropurpurea* and *V. vinifera* (Figure S6, Supporting Information), indicating that no recent whole‐genome duplication (WGD) events occurred in the *M. atropurpurea* genome. The fourfold degenerate transversion rate (4DT) and *Ks* values also revealed no recent independent WGD events in *M. atropurpurea* (Figure S7, Supporting Information). We found 41 gene families among the 18 722 genes that were specific to the *M. atropurpurea* genome (Figure S8, Supporting Information). Comparison of the differences between the species and ancestor revealed that 272 gene families underwent expansion and 1213 gene families underwent contraction in *M. atropurpurea* (Figure 1c). Gene Ontology (GO) enrichment analysis showed that the significantly expanded gene families of *M. atropurpurea* were mainly enriched in organic cyclic compound binding, ion binding, and transferase activity, whereas the contraction genes were enriched in transferring alkyl groups, transferring acyl groups, and nutrient reservoir activity (Figure S9, Supporting Information). Kyoto Encyclopedia of Genes and Genomes (KEGG) enrichment analysis showed that the expanded gene families were significantly enriched in plant‐pathogen interactions, circadian rhythm, and flavonoid biosynthesis, which were mainly involved in sulfur metabolism, ubiquinone biosynthesis, and cysteine and methionine metabolism (Figure S9, Supporting Information). Detailed annotations of the expansion and contraction gene families are listed (Data S6 and S7, Supporting Information).

### 2.2 Basic Population Genetic Characteristics within the Mulberry Tree

To gain further insights into the genomic variation of the mulberry cultivar, we re‐sequenced 290 mulberry accessions with an average depth of 20× per accession (including 276 accessions from 18 provinces in China across major mulberry‐growing areas and 14 accessions from six countries (Thailand, India, Vietnam, Japan, Azerbaijan, and Argentina)) and combined 135 accessions from Jiao et al.^[^ ^1^ ^6]^ and *M. notabilis*.^[^ ^21^ ^]^ A total of 10 998 million clean reads from 425 mulberry accessions were used for the analysis, and 97.13% of the reads on average were mapped onto the referenced assembled *M. atropurpurea* genome (Figure 3a and Data S1, Supporting Information). We identified 2 359 117 high‐quality single nucleotide polymorphism (SNP) and 934 187 indels (length <10 bp) in 425 accessions (Table S8, Supporting Information). Among these, 215 622 SNPs (11.88%) and 16 134 indels (1.73%) were located in the coding regions. SnpEff analysis identified 115 791 non‐synonymous SNPs (4.91%) and 10 175 (0.43%) frame‐shifted indels.

**Figure 2:**
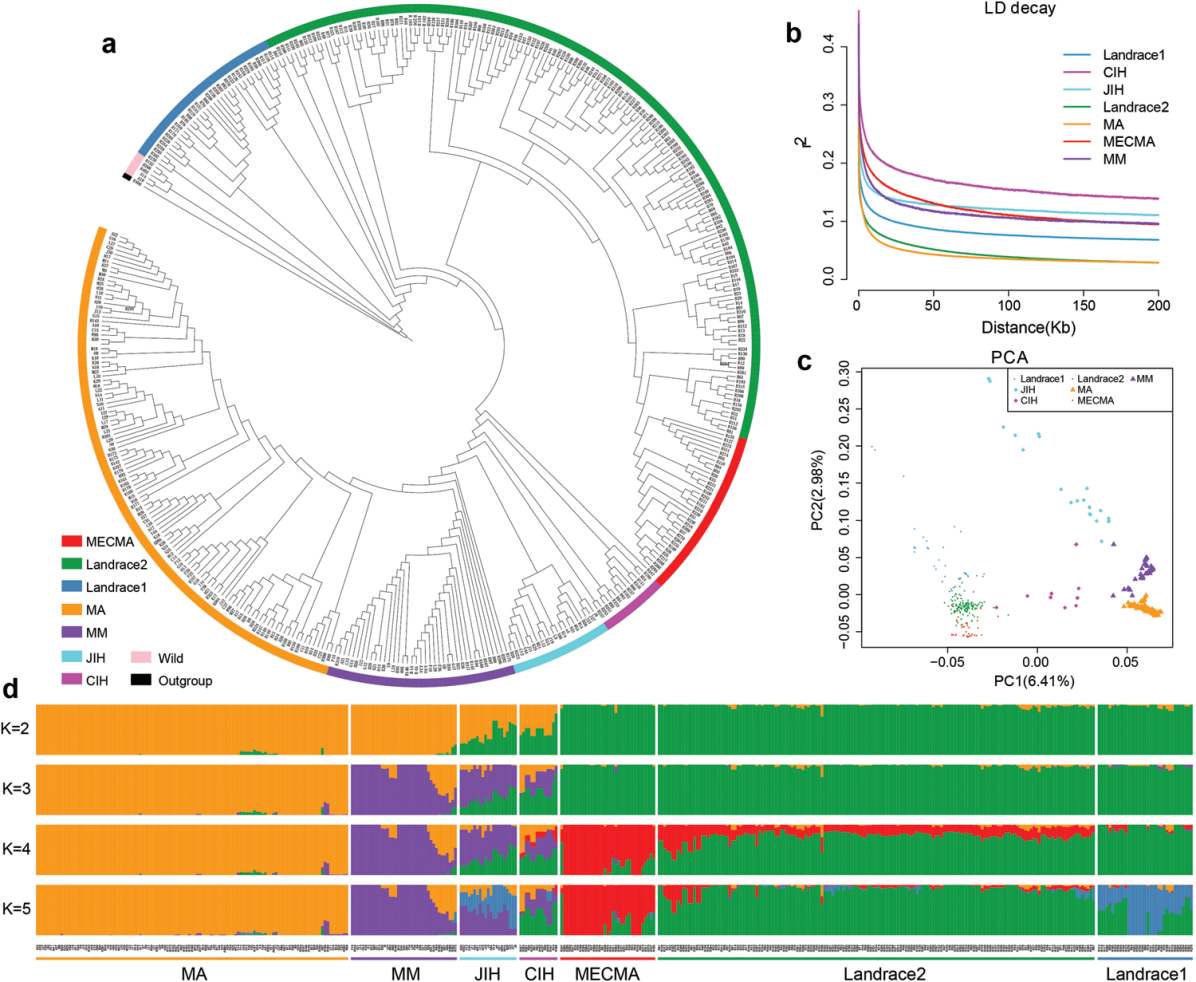
Phylogenetic relationships and population structure of 425 resequenced mulberry accessions. a) Phylogenetic tree of mulberry accessions. *Cannabis sativa* was used as an outgroup. Color codes of accessions are consistent throughout Figure a–d (orange, *M. alba* (MA); purple, *M. multicaulis* (MM); brilliant blue, interspecific hybrid from Japan (JIH); pink, interspecific hybrid from China (CIH); red, modern elite cultivars *M. atropurpurea* (MECMA); green, Landrace2; dusty blue, Landrace1; beige color, wild). b) LD decay‐distance analysis. c) Principal component analysis (PCA) plots of the mulberry populations. d) Population structure based on different numbers of clusters (*K* = 2–5). The *x* axis indicates the mulberry groups with all accessions arranged in the same order as in a. The left *y* axis quantifies genetic diversity in each accession, which is represented by a vertical color‐coded column.

**Figure 3:**
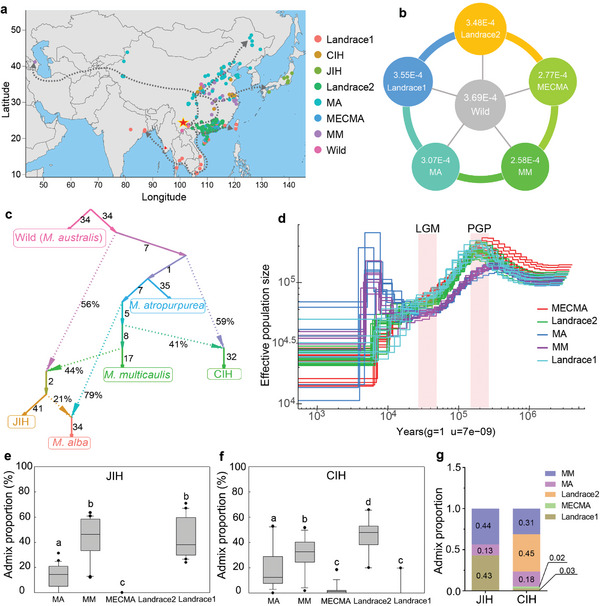
Demographic history of mulberry. a) Geographical distribution of mulberry accessions used in this study. Arrows indicate potential dispersal routes of mulberry. The red star indicates southwest China as a potential origin region of domesticated *M. atropurpurea*. b) Nucleotide diversity (Pi) among six different mulberry groups. The numbers within the circles represent the mean value of nucleotide diversity in each group. ANOVA analysis using LSD and Dunnett's T3 tests showed that significant difference (*p* < 0.05) was detected in each pairwise comparison except Landrace1 versus Landrace2 (*p* = 0.620) in Pi. c) Phylogenetic tree and genetic drift between different species using the f3‐statistic method. The number on the branch indicates branch length and the percentage indicates the proportion of admixture. d) Historical effective population size of mulberry. The shaded pink column represents the time interval of the LGM and PGP. LGM, Last Glacial Maximum (26.5–19 KYA). PGP, Penultimate Glacial Period (130–115 KYA). e–g) Admix proportion of accessions from interspecific hybrids in e) JIH (interspecific hybrid from Japan) group and f) CIH (interspecific hybrid from China). Different lowercase letters indicate a significant difference between each group (LSD and Dunnett's T3, *p* < 0.05). g) Average admix proportion of JIH and CIH from different mulberry subpopulations. MA (*Morus alba*), MM (*M. multicaulis*), and *M. atropurpurea* (including Landrace1, Landrace 2, and MECMA (elite cultivars) subgroups).

To understand the genetic basis of mulberry populations, we constructed a phylogenetic tree using SNPs from 425 mulberry accessions. *C. sativa* was used as the outgroup. The phylogenetic tree divided all accessions into five different genetic groups: Wild, *M. atropurpurea* (including Landrace1, Landrace2, and MECMA (elite cultivars) subgroups), CIH (interspecific hybrid from China), JIH (interspecific hybrid from Japan), and MAM (including MA (*M. alba*) and MM (*M. multicaulis*) subgroups) (Figure 2a). For genetic structure analysis, K values from 1 to 12 and the corresponding cross‐validation (CV) error values were also evaluated (Figures S10 and S11, Supporting Information). When the K value was 5, the population genetic divergence was consistent with the results of principal component analysis (PCA), and each group was clearly isolated and supported by a phylogenetic tree (Figure 2). Kinship analysis revealed that it had little effect on the genetic structure of the population (Figure S12, Supporting Information). Notably, phylogenetic tree and admixture analyses revealed that the germplasm from Japan (JIH group) belonged to the interspecific hybrids between *M. atropurpurea* from the Landrace1 group (43%) and MM (44%) or MA (13%) groups, whereas the CIH group was mainly produced by hybridization between *M. atropurpurea* from Landrace2 (45%) and MM (31%) or MA (18%) (Figures 2d and 3e–g). We observed a gradual decrease in nucleotide diversity (Pi) from the Landrace1, Landrace2, and MECMA groups and significant differences (*p* < 0.05) among the pairwise compared groups except for Landrace1 versus Landrace2 (*p* = 0.620) (Figure 3b), suggesting that the reduction of nucleotide diversity was caused by domestication and human selection.

Based on the phylogenetic tree and the geographic sources of the accessions, all cultivated mulberry accessions formed a single monophyletic lineage derived from wild mulberry (Figure 2a). The mulberry accessions in the Landrace1 group were distributed in southern China (Taiwan, Hainan, Guangxi, and Yunnan) and southeast Asian countries, whereas accessions in the Landrace2 and MECMA groups were mainly from Guangxi and Guangdong. Accessions in the MM and MA groups were widely distributed in different provinces in central China and northern China, respectively (Data S1, Supporting Information). In addition, mulberry accessions from southeast Asian countries (Thailand, Vietnam, and India) were clustered with mulberry accessions originating from Yunnan (China) in the Landrace1 group, indicating that mulberries in these areas have a closer relationship (Figure 2a and Figure S13, Supporting Information). Accessions from Korea, Azerbaijan, Ukraine, and Argentina were clustered in the MM and MA groups, which may have dispersed from central or northern China (Figure S13, Supporting Information). Owing to the lack of documentation of mulberry tree plantations in south Asian countries in ancient times, we hypothesized that cultivars of *M. atropurpurea* might be independently domesticated in southern China. However, given the limited sampling of wild accessions, this hypothesis awaits further confirmation with a complete and wide sampling of the populations of its wild relatives. Two potential migration routes of mulberry domestication were tentatively inferred (Figure 3a). One was from south China to Southeast Asia and the other was from Central and Southwest China to East Asia, Europe, and America.

### 2.3 Demographic History of Mulberry Domestication

F3 and ABBA‐BABA statistics identified extensive gene flow among different mulberry species and populations (Figure 3c and Figure S14, Supporting Information), indicating frequent interspecific and intraspecific introgression, which may have been caused by historical germplasm exchanges during mulberry domestication and cultivation. For instance, significant gene flow was detected from Central to Northwest and Southeast in China, from wild mulberry to cultivated mulberry, and from the MM population to the JIH population (Figure S14, Supporting Information). Historical documents^[^ ^25^ ^]^ indicated that some accessions in the CIH group were bred from crossings between *M. atropurpurea* and *M. alba* or *M. multicaulis*, which was supported by population structure analyses (Figure 2d). Furthermore, the f3‐statistic and admixture proportion analysis showed that the hybrids in CIH group were genetically 59% from ancestor of *M. atropurpurea* and 41% from ancestor of *M. multicaulis*; the hybrids in JIH group were genetically 56% from earlier ancestor of *M. atropurpurea* and 44% from ancestor of *M. multicaulis* (Figure 3c). This result was similar to the statistics of the admixture proportion (Figure 3e–g and Data S2, Supporting Information) and supported the results of CIH and JIH from the hybridization of different populations of *M. atropurpurea*. The ABBA‐BABA statistics with significant positive D‐statistics (Z‐score >3 and *p*‐value <0.001) further indicated widespread introgression among the different subpopulations and species during domestication (Table S9, Supporting Information). In addition, significant introgression was observed in different areas during the domestication of mulberry (Figure S14 and Data S3, Supporting Information).

To delve into the demographic history of multiple mulberry species, we used pairwise sequential Markovian coalescence (PSMC) and SMC++ analyses to investigate how the effective population size (*N*_e_) has changed over the evolutionary history of mulberry. A similar trend of *N*_e_ decline was detected by both PSMC and SMC++ and exhibited a lower historical *N*_e_ of MM and MA compared with that of *M. atropurpurea* (Landrace1, Landrace2, and MECMA) (Figure 3d and Figure S15, Supporting Information). The PSMC results revealed that both *M. atropurpurea* and MAM underwent a bottleneck, which started at ≈0.2 MYA and mapped to a known dramatically low temperature of the Quaternary glaciation (2.58 MYA to present) (Figure 3d), consistent with the shrinkage of *N*_e_ during this period in many other plants,^[^ ^26^, ^27^, ^28^ ^]^ which sheds new light on the demographic responses of mulberry to geological and climatological fluctuations (Figure 3d). Notably, species differentiation between *M. atropurpurea* and *M. alba* also occurred during the same period (Figure 1c), indicating that *M. atropurpurea*, *M. alba* or *M. multicaulis* had a common ancestor before the Quaternary glaciation and that their population separation and speciation might have started long before the Penultimate Glacial Period (PGP) (130–115 kilo years ago (KYA)). The *N*_e_ of MAM reached a minimum and then started to rebound at ≈20 KYA until 5 KYA, coinciding with the stable rewarming of the global climate after the extremely low temperatures of the Last Glacial Maximum (LGM) (26.5–19 KYA).^[^ ^29^ ^]^ However, the *N*_e_ of *M. atropurpurea* has continuously decreased in the recent past. The last period of reduction of *N*_e_ in both *M. atropurpurea* and MAM was likely due to the domestication bottleneck, but this requires further confirmation owing to the limited reliability of the *N*_e_ estimate in the recent past using these analysis methods. Taken together, these two clades presented different historical demographics after divergence.

Meanwhile, the demographic history of *M. atropurpurea* accessions from India, Thailand, and Vietnam showed a similar dynamic tendency of *N*_e_ with accessions from China, that is, a continuous reduction of *N*_e_, whereas accessions from Azerbaijan, Argentina, and Japan showed similar *N*_e_ dynamics to the MAM group, that is, an increase in the *N*_e_ post LGM period (Figure S16, Supporting Information), suggesting that accessions from these countries might have experienced similar historical demographics.

### 2.4 Genetic Underpinning of Key Agronomic Traits

**Figure 4:**
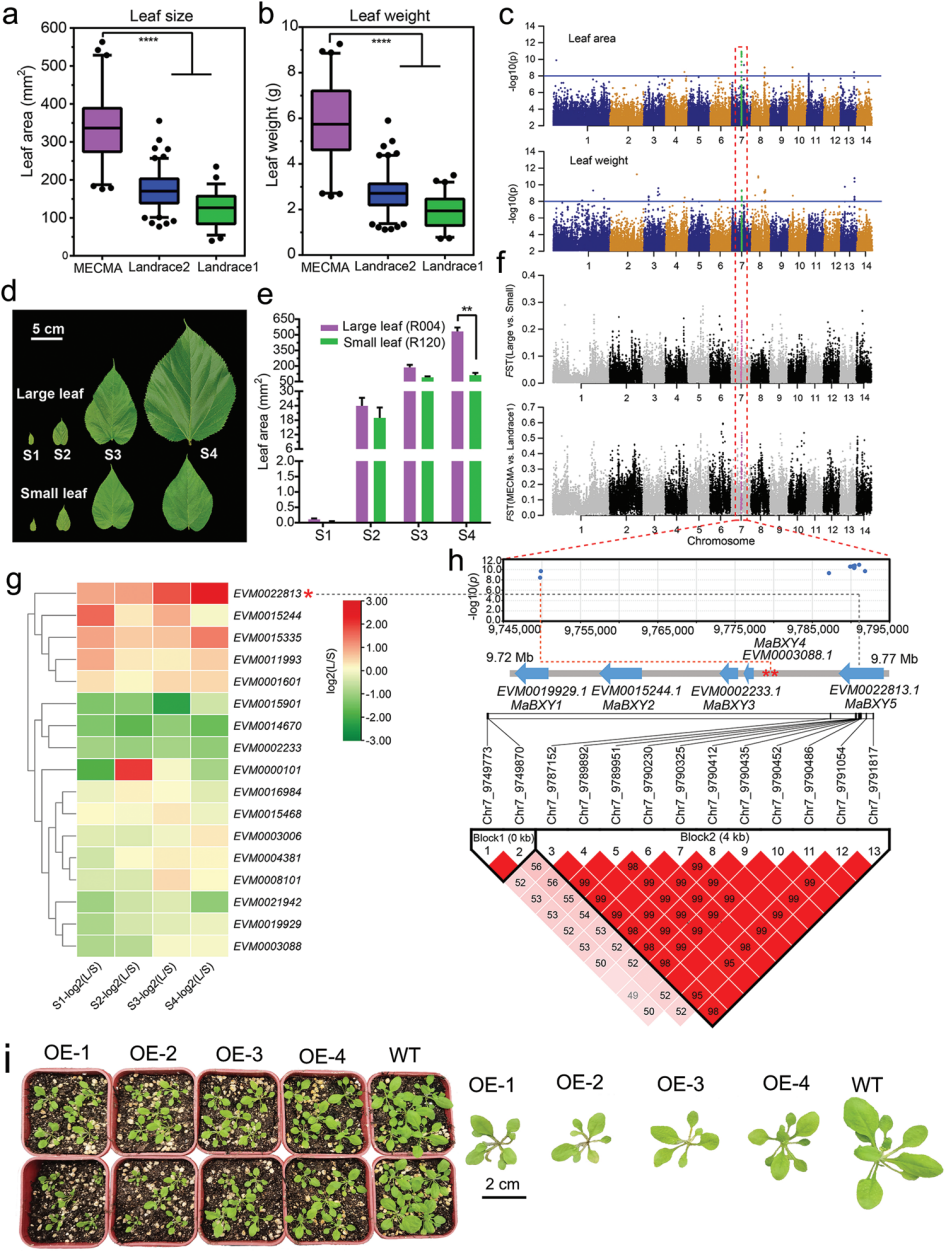
Genomic loci associated with leaf size during *M. atropurpurea* domestication. a,b) Box plots showing significant differences in leaf size (a) and leaf weight (b) between the MECMA and landrace2 and landrace1 groups. The MECMA group with larger leaves is widely cultivated in Guangdong. ** indicates *p* < 0.01 with Student's *t*‐test. c) Manhattan plot of GWAS and f) selective sweeps for leaf size and leaf weight. Significant overlapping loci were identified on chromosome 7 and highlighted by the red dashed column. The blue line indicates significance threshold −log_10_(*p*) ≥ 8 in GWAS. d) Comparison of leaf development in four different stages between large leaf variety (“Tang10”) from the MECMA subgroup and small leaf variety (“Luozhi4”) from the Landrace2 subgroup. e) Bar plot showing significant differences between large‐ and small‐leaf varieties in the S4 development stage (12 d after bud sprouting). ** indicates *p* < 0.01 with Student's *t*‐test. g) Expression pattern of candidate genes across four different leaf development stages. L/S, Large leaf/Small leaf. h) Leaf size‐based association mapping located candidate loci between 9 747 000 and 9 793 000 on chromosome 7 and pairwise linkage disequilibrium (LD) analysis between the significant SNPs. Five genes encoding endo‐1,4‐beta‐xylanase (MaBXY) were tandemly duplicated and indicated by blue arrows (all gene annotations are shown in Table S10, Supporting Information). Pairwise LD block analyses (*r^2^* > 0.9) of SNPs with −log_10_(*p*) ≥ 8. i) Leaf size feature of *MaBXY* transgenic *Arabidopsis* seedlings.

**Figure 5:**
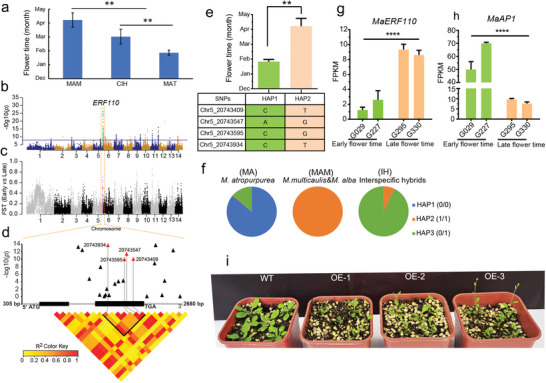
Identification of the *MaERF110* gene associated with flowering time. a) Flowering time in different groups. Statistical significance was determined using a two‐sided *t*‐test. b) Manhattan plot for GWAS on flowering time. The blue line indicates the significance threshold with −log_10_(*p*) ≥ 8. c) Selection signatures by calculating genetic differentiation (*F*_ST_) between early and late flowering accessions across the genome in 100‐kb sliding windows with a step size of 20 kb. The orange dashed column indicates significant SNPs overlapping with GWAS hits, which located the *MaERF110* gene. d) *MaERF110*‐based association mapping and pairwise LD analysis. Triangles show SNPs within the *MaERF110* gene. Haplotype SNPs in the coding region have large effects and were highlighted in red. Strong LD with the lead SNP is connected to the pairwise LD with grey solid lines. e) Haplotypes (Hap) of *MaERF110* among mulberry varieties. Statistical significance was determined using a two‐sided *t*‐test. The flowering time distribution of each haplotype group is displayed as a bar plot. f) Proportion of haplotypes of *MaERF110* in different mulberry groups. g) Expression levels of *MaERF110* in two early flowering time mulberry trees and two late flowering time mulberry trees. h) Expression levels of *MaAP1* in flowering tissues from early and late flowering time mulberry trees. Data are presented as the mean ± S.E. (*n* = 3 independent RNA‐seq experiments). FPKM, fragments per kilobase per million reads. i) Flower time feature of *MaERF110* transgenic *Arabidopsis* seedlings.

The domestication, improvement, and geographic expansion of mulberry involve key transitions in several traits, including the expanded leaf size and biomass in the elite cultivars compared with the early domesticates and landraces of *M. atropurpurea* (Figure 4a,b) and the late flowering time when expanding from southern to central and northern China (Figure **5**a). The flowering time of MAM is obviously later than that of *M. atropurpurea*, even though they are planted under the same environment (Figure 5f).^[^ ^17^, ^25^ ^]^ The leaf size of *M. atropurpurea* is larger than that of the MAM. In addition, the fruit of MAM is white or black, whereas that of *M. atropurpurea* is black.^[^ ^17^, ^25^ ^]^

Leaf quality and biomass are important agronomic traits in silkworm forage and silk production in sericulture. Compared with the Landrace1 and Landrace2 accessions, the elite‐cultivated mulberry (MECMA) had larger leaf size and weight (Figure 4a,b). Leaf size showed significant differences at the S4 developmental stage (12 days after bud sprouting) (Figure 4d,e). To detect genomic loci controlling leaf size and biomass in mulberry, we evaluated the genetic divergence between extremely large and small leaf varieties and between the Landrace1 and MECMA groups. The *F*_ST_ analyses in the two contrast groups consistently revealed a significantly selected signature on chromosome 7, which was further supported by loci correlated with leaf size and biomass in the genome‐wide association study (GWAS) analyses (Figure 4c,f and Figure S17a–b, Supporting Information). A total of 13 significantly associated SNPs (−log10(*p*) > 8) were identified in the 9.72–9.83 Mb region harboring 17 protein‐coding genes (Tables S10 and S11, Supporting Information). Most of these genes are related to carbohydrate metabolism, which has been reported to greatly affect leaf development in grapes.^[^ ^30^ ^]^ Interestingly, five genes encoding endo‐1,4‐beta‐xylanase (*MaBXY*) were duplicated in tandem on the chromosome and were defined as *MaBXY1* to *MaBXY5* (Figure 4h). Among these genes, *EVM0022813 (MaBXY5)* was more than fourfold more highly expressed at the S4 stage in large‐leaf accessions than in small‐leaf accessions (Figure 4g). Moreover, two SNPs with the lowest *p* values were located downstream of the *MaBXY5* gene (Figure 4h), which was annotated as an endo‐1,4‐beta‐xylanase and identified as a key candidate gene controlling leaf size in grape.^[^ ^30^ ^]^ Overexpression of *MaBXY5* affected the leaf size and biomass of *Arabidopsis* (Figure 4i), suggesting that it is a key candidate gene involved in the regulation of mulberry leaf size.

To detect genomic loci controlling flowering time, we first conducted GWAS analyses, unveiling a strong association peak located at 20.69–20.77 Mb region on chromosome 5, which was also a signature of selective sweep in the *F*_ST_ analyses between the *M. atropurpurea* and MAM groups (Figure 5b,c and Figure S17c, Supporting Information). This region included 66 significant SNPs (−log10(*p*) ≥ 8) and five candidate genes (Figure 5b and Tables S12 and S13, Supporting Information). Among these significant SNPs, four with large effects were located in the second exon of the *EVM0010692* gene, which encodes an ethylene‐responsive transcription factor (Figure 5d and Table S12, Supporting Information) that shares 77% protein sequence identity with *AtERF110* in *Arabidopsis*.^[^ ^31^ ^]^ Therefore, we designated it as *MaERF110*. Moreover, we also identified 32 indels located in *MaERF110*, four of which were annotated as codon indels, but only one (Chr5:20 743 943) was correlated with flowering time (Figure S18 and Table S14, Supporting Information).

We subsequently extracted sequences of *MaERF110* from 32 early‐and 29 late‐flowering accessions. The 61 mulberry accessions were classified into two haplotype groups based on the four SNPs derived from *MaERF110* (Figure 5e). Accessions with haplotype 1 showed significantly earlier flowering times than accessions with haplotype 2 and were designated early‐ and late‐flowering *MaERF110* haplotypes, respectively (Figure 5e,f). The expression level of *MaERF110* in flower buds was positively correlated with flowering time (Figure 5g). Because the expression level of the *ERF110* isoform was consistently associated with the expression of a positive integrator of the flowering signaling gene, *APETALA1* (*AP1*), in *Arabidopsis*,^[^ ^31^, ^32^ ^]^ we also examined the expression of the *MaAP1* gene and found that it was significantly downregulated (more than fivefold) in late‐flowering accessions (Figure 5h). Overexpression of *MaERF110* affected the flowering time of *Arabidopsis* plants (Figure 5i). These results indicated that the ethylene‐responsive transcription factor *MaERF110* is a key candidate gene associated with flowering time in mulberry.

### 2.5 Genomic Signatures of Sex Determination in Mulberry

**Figure 6:**
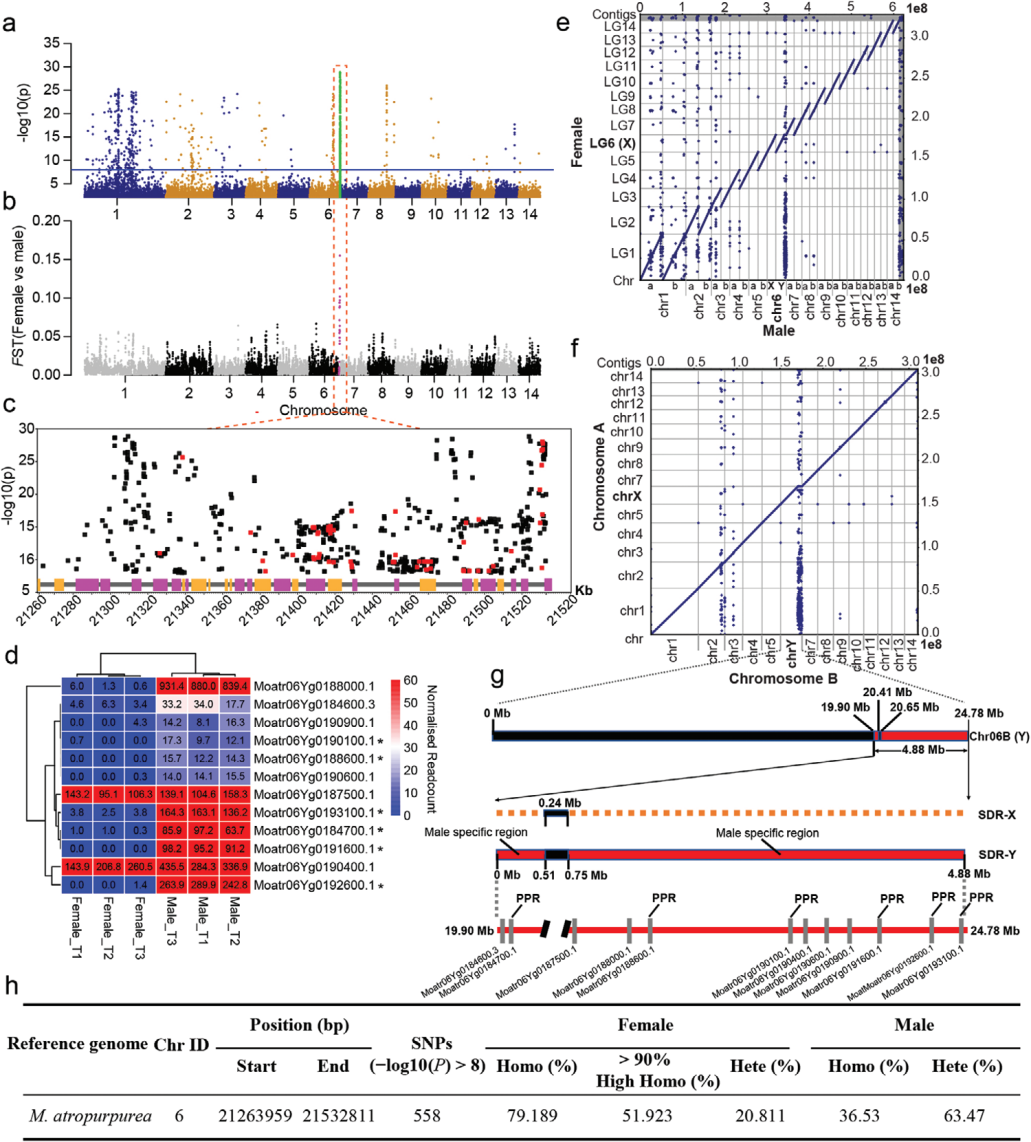
Sex determination region on chromosome 6 in *M. atropurpurea*. a) Manhattan plot of the results of GWAS for sex determination with the female genome “Tang 10” as a reference. The blue line indicates the significance of associated SNPs with a threshold value of −log_10_(*P*) ≥ 8. b) Genetic differentiation (*F*_ST_) between female and male trees across the genome based on the *F*_ST_ calculation of SNPs in 100‐kb sliding windows with a step size of 20 kb. The orange dashed column indicates the peaks the of sex divergence region overlapping with the peak of GWAS. c) Sex‐based association mapping located significant loci between 21 260 000 and 21 530 000 on chromosome 6. The squares represent SNPs (−log_10_(*P*) ≥ 8) across the sex determination region, and nonsynonymous SNPs are highlighted in red. The candidate genes are indicated by purple (forward) and yellow (reverse) boxes. Functional annotation on these genes is shown in Table S17, Supporting Information. d) Gene expression levels of candidate genes located in SDR‐Y region. T1, stage of dormant bud sprouting; T2, stage of early axillary bud; T23, stage of late axillary bud. The star indicates the pentatricopeptide repeat gene (PPR). e) Whole‐genome alignments of each chromosome in the male reference against the female *M. atropurpurea* “Tang 10” reference. f) Whole‐genome alignments of chromosome A against the chromosome B in male *M. atropurpurea* “Huiqiu1.” g) Genome structure plot showing male‐to‐female differences in SDR and the distribution of male specifically genes across the Y‐SDR region. PPR indicates pentatricopeptide repeat gene. h) Statistics of the heterozygosity level of the 558 sex associated SNPs (*p* value < 1e‐8). Male individuals have higher heterozygosity than female.

*M. atropurpurea* accessions can be either dioecious or monoecious, and the genetic mechanism of their sex determination (SD) region remains unclear. A GWAS integrating 70 male and 104 female individuals of *M. atropurpurea* identified an apparent peak at the end of chromosome 6 associated with SD (Figure **6**a and Figure S17d, Supporting Information), and a total of 558 significant SNPs (−log_10_(*P*) > 8) were detected, which explained, on average, more than 30% of the phenotypic variance (Data S4, Supporting Information). ≈80% of the 558 significant SNPs were homozygous in all females, while only 36.53% of them were homozygous in all males (Figure 6h), supporting previous studies that the sex determination mode of mulberry belonged to the XY system.^[^ ^33^, ^34^ ^]^ The population genetic differentiation index *F*_ST_ between randomly selected male (60) and female (60) accessions (Data S5, Supporting Information) showed a single peak at the end of chromosome 6 (Figure 6b), further supporting the GWAS results. The location of the SD region at the telomeres of chromosomes has also been reported in *Populus* ^[^ ^35^ ^]^ and banyan (*Ficus benghalensis*).^[^ ^36^ ^]^ To further understand the genome structural variation of SD region, we aligned the female and male genomes and identified an obvious structural variant at the end of chromosome 6 (Figure 6e–f). We identified the SD region for the Y haplotype (SDR‐Y) as 4.88 Mb assembled reads, which was much longer than the SD region of X haplotype (SDR‐X) (0.24 Mb) (Figure 6g). Annotation predicted that 42 genes were specifically located in SDR‐Y, of which only 12 were expressed (Figure 6g and Tables S15 and S16, Supporting Information). A cluster of six tandem genes encoding a pentatricopeptide repeat‐containing protein (PPR) was identified in SDR‐Y and was specifically expressed at different stages in male plants (Figure 6d). Several PPR proteins have been shown to play important roles in male sterility systems and sex determination^[^ ^37^, ^38^ ^]^ in rice,^[^ ^39^, ^40^ ^]^ strawberry,^[^ ^41^ ^]^ and *Mimulus guttatus*.^[^ ^42^ ^]^ Our results also suggest that PPRs in the SD region are putative candidate genes involved in sexual regulation in *M. atropurpurea*.

## 3 Discussion

Here, we assessed the genomic diversity, domestication, and dispersal of mulberry populations using whole‐genome resequencing data from 425 mulberry accessions, including neglected accessions from Southern China from previous reports,^[^ ^16^ ^]^ which revealed the complete evolutionary history of mulberry. Our genomic diversity and population structure analyses revealed that *M. alba* and *M. multicaulis* should be treated as one species because they are phylogenetically intertwined. All samples of Hu mulberry (geographical group named Taihu Basin of southeastern China by Jiao et al.^[^ ^16^ ^]^) were *M. multicaulis*, and all samples of NH mulberry (non‐Taihu Basin from north and southwest China described by Jiao et al.^[^ ^16^ ^]^) were *M. alba* (Figure S13, Supporting Information). Moreover, we found that accessions from Japan published by Jiao et al.^[^ ^16^ ^]^ were derived from a cross between *M. atropurpurea* in the Landrace1 group and MAM. Furthermore, we identified the key candidate genes controlling leaf size/biomass, flowering time, and sex determination in mulberry. Our study not only provides a holistic view of the domestication and expansion history, but also identifies crucial candidate genes for the future improvement of mulberry.

Comprehensive sampling of accessions enabled us to investigate the domestication, expansion, and introgression of mulberry, providing unique insights into the evolutionary history of economically important perennial species. First, as *M. atropurpurea* and *M. alba* species diverged ≈5.6 MYA and formed monophyletic groups (Figures 1c and 2a), largely predating the domestication time of mulberry, we inferred that *M. atropurpurea* and *M. alba* were possibly domesticated independently from different wild relatives. Five wild species (three accessions from southwest China and two accessions from north China) were clustered together and located at the base of the phylogenetic tree (Figure 2a and Figure S13, Supporting Information), further supporting the hypothesis that *M. alba* and *M. atropurpurea* might have been independently domesticated from two different mulberry progenitors originating from north and south China, respectively. Such independent domestication events of tree crops in different climatic regions have been reported for pears^[^ ^43^ ^]^ and apricots.^[^ ^44^ ^]^ For elite cultivars of *M. atropurpurea*, the reduction of genetic diversity during domestication was 24.3% (calculated as (3.69–2.77)/3.69; see Figure 3b), which is greater than 17% in maize,^[^ ^45^ ^]^ but smaller than 80%–90% in rice^[^ ^46^ ^]^ and 69%–84% in wheat.^[^ ^47^ ^]^ The reduction in genetic diversity during mulberry domestication is consistent with expectations caused by its long‐term domestication history and human selection for silkworm feed purposes. Second, the phylogenetic tree and demographic analyses revealed that mulberries from southeast Asian countries were clustered with *M. atropurpurea* and most likely spread from south or southwest China, whereas mulberries from European and American countries were clustered with *M. alba* or *M. multicaulis* and probably spread from central or northern China (Figure S13, Supporting Information). Integrating knowledge from historical documents, we inferred that *M. atropurpurea* might have dispersed into Southeast Asian countries through the Red River Basin/Mekong River Basin, which was the main transportation route between southern China and southeast Asia in ancient times.^[^ ^48^ ^]^ However, *M. alba* and *M. multicaulis* were introduced from central or northern China to Europe and America via ancient silk roads around the first year A. D.^[^ ^49^ ^]^ Third, the genetic source of hybrids in the JIH group was 43% from *M. atropurpurea* (Landrace1), 44% from *M. multicaulis*, and 13% from *M. alba*, whereas it was 45% from *M. atropurpurea* (Landrace2), 31% from *M. multicaulis*, and 18% from *M. alba*. We inferred that the current hybrids might be the offspring of F_1_ backcrossed with *M. atropurpurea* and rendered by further evolutionary forces, such as drift, selection, and introgression, reshuffling genomic diversity. In addition, extensive gene flow was observed among mulberry populations, with particularly strong signals of introgression between cultivated mulberry in Yunnan and those from other geographical regions (Table S9 and Data S3, Supporting Information), implying that pervasive introgression events play a significant role in perennial domestication.^[^ ^7^, ^9^ ^]^

Leaf agronomic traits related to leaves are vital domestication targets for many vegetables. For instance, the *FAR1* gene on chromosome 1 has been identified as a potential candidate for organ size control during spinach domestication.^[^ ^50^ ^]^ Modern lettuce cultivars display differentiated characteristics in leaf development traits compared to their wild relatives, and TCP transcription factor proteins were detected as promising candidates associated with leaf margin undulation.^[^ ^51^ ^]^ However, existing studies have only described the genetic mechanisms underlying leaf development in a few annual crops. Leaf size in mulberry is also a typical agronomic trait that underwent long‐term artificial selection for the purpose of feeding silkworms at least 5000 years ago, presenting a system to delve into the genetic mechanisms controlling leaf size in perennials. In this study, we found that the elite cultivar *M. atropurpurea* (MECMA) showed a larger leaf size than the landraces, and a hotspot locus located on chromosome 7 containing five tandem duplicated *endo‐1,4‐beta‐xylanases* (*BXYs*) genes was detected as a signature of differentiation during the improvement and was significantly associated with leaf size and biomass. *BXY*s in plants belong to the glycoside hydrolase family 10 (GH10) and play a vital role in plant development and boost primary growth (such as leaf enlargement and stem elongation).^[^ ^52^ ^]^ In grape, *BXY*s are associated with carbohydrate metabolism and affect leaf size and development.^[^ ^30^ ^]^ In summary, our study identified new candidate genes that control leaf size and biomass in perennial species.

Flowering time is a typical domestication trait that has been extensively studied in plants. Two *pseudoresponse‐regulator* (*PRR*) genes control flowering time and maturity during soybean domestication.^[^ ^53^ ^]^ *FLOWERING LOCUS T* (*FT*) paralogs play key roles in controlling flowering time during sunflower domestication.^[^ ^54^ ^]^ The flowering time of mulberry shows substantial changes during dispersal from southern China (flowering in January) to central and northern China (flowering from March to April). Delayed flowering enables mulberry to escape freeze or chill damage to flowering organs and adapt to high‐latitude environments with varied temperatures and photoperiods. By combining the GWAS and *F*_ST_ analysis results, we identified a gene (*MaERF110*) on chromosome 5 that is associated with flowering time variation. Overexpression of the orthologs of *ERF110* in *Arabidopsis thaliana* and *Chrysanthemum morifolium* results in accelerated flowering by operating the circadian clock.^[^ ^31^, ^32^, ^55^ ^]^ Consistent with the gene structure in *Chrysanthemum*, *MaERF110* in mulberry also included a conserved DNA‐binding AP2/ERF domain (Figure S18b, Supporting Information). However, the expression regulation mode of *MaERF110* in mulberry was distinct from that in *Chrysanthemum*, that is late‐flowering mulberry showed a higher expression level of *ERF110* than early flowering mulberry. This finding suggests a new regulatory interaction of *MaERF110* in flowering time regulation in mulberry compared to that in *Chrysanthemum*. Reports have shown that genes undergoing rapid changes in sequence, structure, and expression may indicate the origin of new genes or new gene functions.^[^ ^56^ ^]^ For example, allele‐specific expression variations in *Capsella grandiflora* have revealed that alleles affecting gene expression are rare and exhibit a negative correlation between their frequency and phenotype.^[^ ^57^ ^]^ Thus, we inferred that these extensive variations (more than 32 variants) in *MaERF110* probably altered the function of its regulatory elements and affected gene expression and regulation of flowering time. Further molecular experiments are required to explore the relationships between genetic variation, gene expression of *MaERF110*, and flowering time.

GWAS and selective sweep analyses showed significantly overlapping signals in the SD region on the proximal telomeric end of chromosome 6. We also found an apparent segregation of heterozygous and homozygous genotypes of SNPs in this region between males and females, although the differential frequency of heterozygous and homozygous genotypes of SNPs between males and females was not always an obvious sign of recombination suppression. This phenomenon has also been observed in other species. For example, *Populus qiongdaoensis* exhibits ZW sex determination on chromosome 19; 68% of the genotypes are heterozygous in females, whereas ≈80% of the genotypes are homozygous in males.^[^ ^58^ ^]^ The mulberry SD region also has similar proportion of heterozygosity (63.5%) and homozygosity (79.2%) in this study (Figure 6e). A recent study reported that an ≈6.2 Mb SD region on chromosome 3 was characterized in *M. notabilis* based on short Illumina read analysis of four male and four female plants of *M. notabilis*.^[^ ^18^ ^]^ In *M. atropurpurea*, we found a 4.88 Mb SDR‐Y region on chromosome 6B. Syntenic block analysis showed that it corresponds to the SD region in other mulberry species^[^ ^18^ ^]^ (Figure S5, Supporting Information). This implies a potentially similar SD mechanism among mulberry species, although the SD region is located on different chromosomes.

Based on transcriptome analysis, genomic variation, and gene functional annotation, we identified 30 functional candidate genes potentially involved in SD in the GWAS‐significant regions (Table S17, Supporting Information). Four of them (FT gene (*EVM0020947.1*), GDSL esterase/lipase gene (*EVM0018167.1*), *CYP79B2* (*EVM0012542.1*), and inositol‐polyphosphate 5‐phosphatase (*EVM0014931.1*)) were reported to be associated with flower and pollen development. The *FT* gene is located in sex‐specific genomic regions and is specifically expressed in male bayberry (*Morella rubra*).^[^ ^59^ ^]^ However, *FT* genes were not highly expressed in male or female plants (Table S18, Supporting Information). The GDSL esterase/lipase gene is essential for rice pollen development^[^ ^60^ ^]^ and is specifically expressed at the dormant bud sprouting stage in males (Table S18, Supporting Information). During flower development, GDSL‐type lipase/esterase genes can be downregulated by DELLA proteins, which are gibberellin (GA) signaling repressors.^[^ ^61^ ^]^ The sex of mulberry can be altered by the application of ethrel and silver.^[^ ^62^ ^]^ Different concentrations of ethylene produced female, male, and mixed inflorescences in male mulberry, whereas the induction of male, female, and mixed inflorescences was observed in female plants after silver nitrate application.^[^ ^62^ ^]^ This indicates that hormones and external environmental stimulation influence plant sex differentiation by affecting the expression levels of SD genes in mulberry.^[^ ^62^, ^63^, ^64^ ^]^ Ethylene and auxin promote female flower formation, whereas GAs promote male flower formation.^[^ ^65^, ^66^ ^]^ In this study, the GDSL esterase/lipase gene (*EVM0018167.1*) was highly expressed (FPKM = 190) in male mulberry, and may be a putative key gene that regulates gas production and is responsible for male flower differentiation. *Cytochrome P450* is considered a male activator in the date palm (*Phoenix dactylifera*),^[^ ^67^ ^]^ which has two copies in the sex determination region of *M. atropurpurea*. *CYP79B2* (*EVM0012542*) was specifically expressed in female mulberry (Table S18, Supporting Information). One study indicated that *CYP79B2* could promote Trp to indole‐3‐acetaldoxime (IOAx) biosynthesis in gynoecious inflorescences rather than in monoecious inflorescence.^[^ ^68^ ^]^ Thus, we suggest that CYP79B2 (*EVM0012542.1*) is a putative candidate involved in female flower differentiation. The candidate inositol‐polyphosphate gene has also been reported to be associated with flower and pollen development.^[^ ^69^ ^]^ Recently, a candidate SD region of *M. notabilis*, including 404 genes, was identified,^[^ ^18^ ^]^ and three male‐specific Ty3_Gypsy retro‐transposon (RT) genes and one male‐specific DNA helicase gene (MSDH) were expressed at higher levels in male flowers. Further exploration of sex‐related genes and regulatory mechanisms is required.

## 4 Conclusion

The current study reports the chromosome‐level mulberry genome and provides novel insights into its evolution. A high‐resolution genomic variation map of 425 mulberry accessions was generated, which provided insights into mulberry classification, gene introgression, and domestication. In addition, several QTL loci and candidate genes that contribute to flowering time, leaf size, and sex determination were identified, offering a valuable genetic basis for improving key agronomic traits.

## 5 Experimental Section

### Sample Preparation

The cultivars “Huiqiu1” and “Tang10” (*Morus atropurpurea*) were widely planted in Guangdong, China. “Huiqiu1” is a male *M. atropurpurea* variety. “Tang10” is an elite female *M. atropurpurea* variety. The two varieties had excellent agronomic traits, including vigorous growth, high yield, well‐developed root system, and wide adaptability. “Huiqiu1” and “Tang10” were used for genome assembly. In total, 290 mulberry accessions from nearly all mulberry plantation regions in China and other countries (Japan, Thailand, India, Vietnam, Azerbaijan, and Argentina) were collected for whole‐genome resequencing (Data S1, Supporting Information). Scions were obtained from the mulberry preservation units of the Guangdong Academy of Agricultural Sciences in Guangzhou, China. Young leaf samples were collected, immediately frozen in liquid nitrogen, and stored at −80 °C for DNA extraction.

### Genome Size Estimation

To estimate the genome size, the DNA of the samples was quantified using flow cytometry (BD, FACSCalibur, CA, USA). Leaf material was prepared and stained with propidium iodide according to a previously reported protocol.^[^ ^70^ ^]^ The ratio of the mean fluorescence of the mulberry G0/G1 peak to that of maize (B37; genome size, 2.3 Gb) was calculated.^[^ ^71^ ^]^ Furthermore, the genome size was evaluated more precisely using the k‐mer distribution of the resequencing reads. A total of 41.41 Gb reads were used for the analyses of k‐mer frequency distribution. The value of K was set to 19. The values of the filtered k‐mers were 31, 953, 256, and 456, respectively, and the major peak depths were 103. Genome size was estimated using the formula Kmer_Number/Peak_depth.^[^ ^72^ ^]^

### Library Preparation and Sequencing

For PacBio sequencing of “Huiqiu1,” the BluePippin system was first applied for size selection. SMRTbell libraries (30–50 kb) were constructed according to the protocol provided by PacBio. Three single‐molecule real‐time cells were sequenced using a PacBio Sequel II platform.

Genomic DNA of “Tang10” was extracted and sequenced according to the manual of the Ligation Sequencing Kit (Nanopore, Oxford, UK). Briefly, DNA was purified and assessed using a Qubit 2.0 Fluorometer (Thermo Fisher, CA, USA). DNA fragments of ≈20 kb were enriched and purified by random shearing with a Covaris g‐TUBE. After the ends were repaired and the adapters were ligated, a 20‐kb library was constructed for sequencing using flow cells on the Nanopore PromethION platform (Nanopore).The Hi‐C sample library was constructed as previously described.^[^ ^73^ ^]^ Briefly, tissue cells and DNA were fixed and digested with formaldehyde and the restriction endonuclease *Hin*d III, respectively. The 5′ overhang of the fragments was repaired, labeled, and ligated into a small volume. The ligated DNA was purified and sheared to a length of 300–700 bp. Finally, the purified DNA fragments were captured using streptavidin beads and sequenced on an Illumina HiSeq X Ten platform (Illumina, San Diego, CA, USA).

Young leaves were collected from mulberry plants for resequencing. Fresh leaves were used to estimate ploidy levels by flow cytometry, using a mulberry variety of known ploidy (TL; 2*x* = 2*n* = 28)^[^ ^74^ ^]^ and maize (B37; genome size 2.3 Gb)^[^ ^71^ ^]^ as external standards. In total, 290 diploid mulberry accessions were used for resequencing. Genomic DNA (>5 µg) for each accession was used to construct paired‐end sequencing libraries with 350 bp insert‐sizes. An average of 20 paired‐end reads for each sample was generated using the Illumina HiSeq 4000 platform.

### Genome Assembly

For male “Huiqiu1,” Primary assembly were generated by two steps: Hifiasm v0.15.4^[^ ^75^ ^]^ was used for assembling the PicBio long‐reads into contigs, followed by dividing the genome into two haplotypes based on Hi‐C data using Hi‐C partition mode. The Hi‐C data were mapped against the primary assembly to obtain a normalized contact matrix with Juicer v1.7.6 (https://github.com/aidenlab/juicer). A Hi‐C contact matrix was used to scaffold the 3D‐DNA pipeline. Subsequently, new scaffolds were obtained with gaps filled by two iterations using TGS_Gapcloser v1.0^[^ ^76^ ^]^ and five rounds of polishing using NextPolish v1.2.4.^[^ ^77^ ^]^ The redundancy of unanchored contigs was removed using Redundans v0.14a (identity >0.98).^[^ ^78^ ^]^

For female “Tang10,” Canu v1.7^[^ ^79^ ^]^ and SMARTdenovo v1.06 (https://github.com/ruanjue/smartdenovo) software were first used to correct the raw Nanopore reads and assemble the error‐corrected reads to obtain the original contigs, respectively. Racon v1.3.1^[^ ^80^ ^]^ and Pilon v1.2.1^[^ ^81^ ^]^ were used to calibrate the error of the draft genome using Nanopore reads to obtain contigs. Hi‐C short reads were mapped to the draft genome to obtain unique read pairs using BWA v0.7.15.^[^ ^22^ ^]^ These unique read pairs were further accessed using HiC‐Pro,^[^ ^82^ ^]^ and invalid interaction pairs were mapped to the draft genome. Finally, all contigs were grouped, sorted, and oriented to obtain chromosome‐level assemblies using the LACHESIS.^[^ ^23^ ^]^ The assembly was further accessed by mapping Illumina short reads, recovery of core eukaryotic genes,^[^ ^83^ ^]^ and core land plant genes from BUSCO v4.0.^[^ ^84^ ^]^

### Repeat Annotation

Repetitive sequences were identified in the *M. atropurpurea* genome by using homology and de novo strategies. De novo prediction software RepeatScout v1.0.5^[^ ^85^ ^]^ and LTR‐FINDER v1.05^[^ ^86^ ^]^ were used to identify repeats within the genome. PASTEClassifier v1.0^[^ ^87^ ^]^ was used to classify these repeats into various categories, which were merged into the RepBase database^[^ ^88^ ^]^ to construct an *M. atropurpurea* genome repetitive sequence database for the identification and annotation of TEs using RepeatMasker v4.0.6.^[^ ^89^ ^]^

### Gene Prediction and Functional Annotation

De novo, homologous, and transcriptome‐based strategies were used for gene model predictions. In the de novo prediction, Genscan,^[^ ^90^ ^]^ Augustus v2.4,^[^ ^91^ ^]^ GlimmerHMM v3.0.4,^[^ ^92^ ^]^ GeneID v1.4,^[^ ^93^ ^]^ and SNAP v2^[^ ^94^ ^]^ were used. For homolog‐based prediction, GeMoMa v1.3.1^[^ ^95^ ^]^ was used. In the transcriptome‐based prediction, HISAT v2.0.4^[^ ^96^ ^]^ and StringTie v1.2.3^[^ ^97^ ^]^ were used to perform reference‐based assembly of the RNA‐seq data. TransDecoder v2.0 (https://github.com/TransDecoder/TransDecoder) and GeneMarkS‐T v5.1^[^ ^98^ ^]^ were used to predict the genes. Unigenes were non‐parametrically predicted using PASA v2.0.2^[^ ^99^ ^]^ based on RNA‐seq data. All predicted results from the different methods were integrated using evidence modeller (EVM) v1.1.1^[^ ^100^ ^]^ and further modified with PASA v2.0.2.^[^ ^99^ ^]^ The predicted genes were annotated and enriched using the Gene Ontology (GO),^[^ ^101^ ^]^ KEGG,^[^ ^102^ ^]^ Eukaryotic orthologous groups (KOG),^[^ ^103^ ^]^ TrEMBL,^[^ ^104^ ^]^ and Nr^[^ ^105^ ^]^ databases based on basic alignment local search tools (BLAST) with an *e* value ≤1 × 10^−5^. In addition, ncRNA (miRNA, rRNA, and tRNA) were predicted using Infenal v1.1^[^ ^106^ ^]^ and tRNAscan‐SE v1.3.1^[^ ^107^ ^]^ according to the Rfam^[^ ^108^ ^]^ and miRbase^[^ ^109^ ^]^ databases. Pseudogenes were predicted using GenBLASTA v1.0.4^[^ ^110^ ^]^ alignment and GeneWise v2.4.1.^[^ ^111^ ^]^

### Evolution Analysis

To investigate the evolutionary history of mulberry, paralogs within the *M. atropurpurea* genome or between mulberry accessions and other species were identified using MCScanX^[^ ^112^ ^]^ based on protein sequence alignments using Diamond v0.9.29.130).^[^ ^113^ ^]^ The 4DT was calculated using the HKY substitution model. The synonymous mutation rate (*K*_S_) was calculated using the WGD program v1.1.1.^[^ ^114^ ^]^ To identify gene family groups, protein‐coding genes were analyzed from 13 species, which included *Arabidopsis* (TAIR10), *Oryza sativa* (MSU_v7.0), *Populus trichocarpa* (v3.0), *Solanum lycopersicum* (SL2.40), *Medicago truncatula* (MedtrA17_4.0), *Vitis vinifera* (12×), *Malus domestica* (HFTH1_v1.0), *Nelumbo nucifera*, *Amborella trichopoda*, *Citrullus lanatus*, *Cannabis sativa*, *Morus notabilis*, *Morus alba*, and *M. atropurpurea* genomes. Orthologous gene groups of *M. atropurpurea* and 13 other species were identified using the OrthoFinder package with default parameters.^[^ ^115^ ^]^ To determine the expansion and contraction of the gene family, the differences in cluster size between species and ancestors were compared using the CAFÉ program v4.2.^[^ ^116^ ^]^ The coding sequences from all single‐copy families were aligned using MAFFT v7.205.^[^ ^117^ ^]^ After removing poorly aligned sequences, well‐aligned sequences were concatenated into supergenes. The best model, JTT+F+I+G4, was determined based on the analysis of ModelFinder^[^ ^118^ ^]^ and used to construct a maximum‐likelihood (ML) phylogenetic tree with 1000 bootstraps. The divergence time among species was estimated using MCMCTREE in PAML v4.9i.^[^ ^119^ ^]^ GO and KEGG annotation of gene family was completed by aligning the genes to the GO and KEGG database and NCBI non‐redundant database using BlastP with an *e* value of 1e−5.

### Genomic Variant Calling and Annotation

After filtering, clean reads were mapped to the reference genome of *M. atropurpurea* using Burrows‐Wheeler Aligner (BWA) v0.7.10.^[^ ^22^ ^]^ The BAM files were processed sequentially for indel realignment, duplicate marking, and base quality recalibration using Picard v2.18 tools (http://picard.sourceforge.net). Calibrated alignments were used to call genomic variants using the HaplotypeCaller Genome Analysis Toolkit (GATK) v4.2.^[^ ^120^ ^]^ The raw SNPs and Indel variants were filtered with the following parameters: “QD < 2.0 || MQ < 40.0 || FS > 60.0 || QUAL < 30.0 ||‐clusterSize 2‐clusterWindowSize 5.” The identified SNPs and indels were further functionally annotated with SnpEff v4.3T tool software.^[^ ^121^ ^]^

### Population Genetic Analyses

To obtain reliable results for the population history of mulberry, SNPs were first filtered by removing those with low MAF (≤0.05) and low genotype rate (≤0.5). To explore the phylogenetic relationships of the 425 mulberry accessions (including the 290 accessions sequenced in this study and 135 accessions from Jiao et al.^[^ ^16^ ^]^ and He et al.^[^ ^21^ ^]^), a rooted phylogenetic tree with *C. sativa* as an outgroup based on the filtered SNPs was constructed using the neighbor‐joining method, with the *P* distance matrix calculated using VCF2Dis (https://github.com/BGI‐shenzhen/VCF2Dis).^[^ ^122^ ^]^ Plink software was used to perform an LD‐pruned selection of SNP loci, and the filtered SNPs were used to infer the population structure using ADMIXTURE v1.3.^[^ ^123^ ^]^ The number of sub‐populations was evaluated using CV method.^[^ ^124^ ^]^ PCA of the SNPs was performed using the smartPCA program with default parameters.^[^ ^125^ ^]^ The first two PCs were used to classify the different groups. LD was calculated between each pair of SNPs using PopLDdecay v3.40.^[^ ^126^ ^]^ The squared correlation coefficient (*r^2^*) values were analyzed using a 1000‐kb window for each chromosome. Genetic differentiation (*F*_ST_) and nucleotide diversity (*π*) were used to detect selective signatures between different groups in 20‐kb non‐overlapping windows using vcftools v.0.1.13.^[^ ^127^ ^]^

To detect genetic introgression between cultivated mulberry plants from different areas and their close relatives, gene flow was investigated using the TreeMix software package^[^ ^128^ ^]^ and calculated the *f*_3_ value using the program ADMIXTOOLS.^[^ ^129^ ^]^ In addition, *ABBA‐BABA* analysis was also performed using the Dsuite program^[^ ^130^ ^]^ to calculate Patterson's *D* statistic, which was widely used to detect genetic introgression in genome alignments for a given four taxa with the relationship “((P1,P2),P3),O.” A *D* statistic value significantly different from zero and Z score (*D*/*std_err*(*D*) > 3 or less than −3 indicates introgression between populations P2 and P3 (*D* value > 0) or between P1 and P3 (*D* value > 0)).^[^ ^131^, ^132^ ^]^

To infer the demographic history of mulberry, a pairwise sequential Markovian coalescent (PSMC) model was used to estimate the historical effective population size, *N*_e_.^[^ ^133^ ^]^ The estimated mulberry mutation rate (7 × 10^−9^) and average generation time of 1 year were used to scale the population parameters into years and individuals. In addition, multigenome analysis for demographic history inference was performed using another sequential Markovian coalescent‐mode, SMC++.^[^ ^134^ ^]^

### Genome‐Wide Association Study

Accessions used for the GWAS in this study were cultivated in field trials in Guangzhou, China, during the 2019 and 2020 growing seasons. Each accession had at least three replicates and was planted with a 1.5‐m distance between plants and 3.5‐m distances between rows. Leaf size and weight were measured in nine mature leaves from at least three trees. Flowering time was defined as the time at when approximately three‐quarters of flowers of the three replicate trees bloomed. Sex phenotypes were investigated every year during the flowering period.

High‐quality SNPs (MAF >0.05, missing rate <0.5) in all mulberry accessions were used to perform a GWAS for four traits (leaf size, leaf weight, flowering time, and sex) using four models: the general linear model (GLM), mixed linear model (MLM), FaST‐LMM, and efficient mixed‐model association expedited (EMMAX).^[^ ^135^, ^136^, ^137^, ^138^ ^]^ PCA was performed using the smartPCA program with default parameters,^[^ ^125^ ^]^ and kinship was calculated using plink with the parameter “–genome ‐min 0.2.”^[^ ^126^ ^]^ Both PCA or population structure and kinship were considered as covariates to reduce the impact of accessions from two different subpopulations or within a subpopulation. For leaf size and weight, all individuals of *M. atropurpurea* were used for the GWAS. For flowering time, 231 *M. atropurpurea*, 18 *M. multicaulis*, and 25 *M. alba* were used for the GWAS analysis. Interspecific hybrids and the two wild‐type individuals were not used. For sex, 70 male and 104 female individuals of *M. atropurpurea* were used for the GWAS analysis. Individuals with bisexual flowers were excluded. The significance threshold was estimated using the Bonferroni multiple testing correction (*α* < 0.05) (approximately *α* log_10_(*P*)) ≥ 8.

### Expression Analysis Using RNA‐seq

For transcriptome analyses, RNA was isolated from samples of the following three groups: The first group was leaf samples comprising two accessions (small leaf: R120/G246; large leaf: R004/G004) at four different leaf developmental stages (Figure 4d); the second group was flower bud samples consisting of four accessions (two early flowering times: R103/G227 and R018/G029; two late‐flowering times: R282/G295 and R160/G330) at the blasting bud stage; and the third group was flower bud samples containing two accessions (female: R103/G227; male: R319/G225) harvested at three developmental stages (T1, stage of dormant bud sprouting; T2, stage of early axillary bud; T3, stage of late axillary bud). Each tissue sample contained three biological replicates.

Total RNA was extracted using the RNeasy Plant Mini kit reagents (Qiagen, Valencia, CA, USA) according to the manufacturer's instructions. A cDNA library was constructed, and sequencing was performed on the HiSeq 2000 platform (Illumina, San Diego, CA, USA), as described previously.^[^ ^27^ ^]^ Data quality and library saturation were analyzed using Illumina/Solexa pipeline software. For the raw data, adaptor sequences were removed using Trimmomatic,^[^ ^139^ ^]^ and clean reads were mapped against annotated gene modules using the Bowtie program^[^ ^140^ ^]^ with the best alignments. The read counts and FPKM values were estimated using RSEM.^[^ ^141^ ^]^ Differentially expressed genes (DEGs) were analyzed using the Noiseq package in R software.^[^ ^142^ ^]^ Fold changes were assessed with false discovery rate (FDR) < 0.05 and fold change (FC) |log2FC| ≥ 1.

### Genetic Transformation of Candidate Genes

To express *MaERF110* and *MaBXY5* in plants, the coding sequences of *MaERF110* and *MaBXY1* were inserted into the *Xba I* and *Kpn I* sites of the binary vector pCAMBIA2300, which contained the *CaMV 35S* promoter, *NOS* terminator, and kanamycin resistance gene. The primers used to detect transgenic plants were NPT‐F: 5′‐AAGATGGATTGCACGCAGGT‐3′ and NPT‐R: 5′‐TCACGGGTAGCCAACGCT‐3′. The resulting plant binary vectors were transferred to *the Agrobacterium tumefaciens* strain GV3101^[^ ^143^ ^]^ and transformed into *A. thaliana* using the floral‐dip method.^[^ ^144^ ^]^

### Statistical Analysis

All statistical analyses were conducted using the SPSS 20.0 (International Business Machines Corporation, Armonk, NY, USA) statistical software package and R software.^[^ ^142^ ^]^ Comparisons between groups for statistical significance were performed with a two‐tailed Student's *t*‐test. Error bars represent mean ± SD derived from three independent experiments. In all cases, *p* value <0.05 was considered statistically extremely significant, and *p* value <0.01 was considered statistically significant. All experiments were performed for at least three times independently under similar conditions.

## Conflict of Interest

The authors declare no conflict of interest.

## Author Contributions

F.D. and X.Z. contributed equally to this work. F.D., X.Z., L.W., Z.Z., Z.–J.L and C.T. designed the experiments and managed the project. F.D., X.Z., T.Z., Z.–J.L., L.W., Z.Z., and C.T. wrote the manuscript with input from all authors. F.D., X.Z., G.L., Z.W., D.W., J.W.Z., S.L., L.C., Z.L., Y.W., T.Z., Z.L., L.W., Z.Z., and C.T. collected the samples, extracted genetic material, analyzed the data, and performed the experiments. F.D., X.Z., G.L., Z.W., D.W., Z.Z., and C.T. performed the experiments and genomic and RNA sequencing. F.D., X.Z., and Z.Z. performed the genome assembly and gene annotation analyses. F.D., J.W.Z., S.L., L.C., Z.L., Y.W., and C.T. performed population and transcriptomic analyses. F.D., X.Z., T.Z., Z.L., L.W., Z.Z., D.Z., Y.L., Q.Z., and C.T. revised the manuscript.

## Supporting information

Supporting Information

Supplemental Table 1

## Data Availability

The data that support the findings of this study are available in the supplementary material of this article.
