# Supplementary material for: Genomic Resequencing Unravels the Genetic Basis of Domestication, Expansion, and Trait Improvement in Morus Atropurpurea
Source: Adv Sci (Weinh). 2023 Jun 20;10(24):2300039. doi: 10.1002/advs.202300039 (PMC10460887; doi:10.1002/advs.202300039)
Supplement: Supplementary file 1 — Supporting Information [file ADVS-10-2300039-s001.pdf]

ADVANCED SCIENCE
Open Access

Supporting Information

for *Adv. Sci.*, DOI 10.1002/advs.202300039

Genomic Resequencing Unravels the Genetic Basis of Domestication, Expansion, and Trait Improvement in *Morus Atropurpurea*

Fanwei Dai, Xiaokang Zhuo, Guoqing Luo, Zhenjiang Wang, Yujuan Xu, Dan Wang, Jianwu Zhong, Sen Lin, Lian Chen, Zhiyi Li, Yuan Wang, Diyang Zhang, Yuanyuan Li, Qinyao Zheng, Tangchun Zheng, Zhong-Jian Liu*, Li Wang*, Zhiyong Zhang* and Cuiming Tang**

Supporting Information

Genomic resequencing unravels the genetic basis of domestication, expansion, and trait improvement in *Morus atropurpurea*

Fanwei Dai[1,7#], Xiaokang Zhuo[2,4#], Guoqing Luo[1,7], Zhenjiang Wang[1,7], Yujuan Xu[1], Dan Wang[1], Jianwu Zhong[1], Sen Lin[1], Lian Chen[1], Zhiyi Li[1], Yuan Wang[1], Diyang Zhang[3], Yuanyuan Li[3], Qinyao Zheng[3], Tangchun Zheng[4]*, Zhong-Jian Liu[3]*, Li Wang[5,8]*, Zhiyong Zhang[6]*, Cuiming Tang[1,7]*

Content

Additional file 1: Figures..4

Figure S1. Analysis of chromosome ploidy of *M. atropurpurea*……….................................4

Figure S2. The flowchart of assembly for the *M. atropurpurea* genome 5

Figure S3. The genome size estimate of *M. atropurpurea*...6

Figure S4. Collinearity analysis of *M. atropurpurea*...7

Figure S5. Chromosome synteny and structural variation between *M. atropurpurea* and *M. alba*.. 8

Figure S6. Schematic representation of syntenies among *M. atropurpurea*, *Populus trichocarpa*, and grape genomes..9

Figure S7. Density distribution of (a) 4DTv (fourfold synonymous third-codon transversion) and (b) Ks for paralogous genes in *M. atropurpurea*..10

Figure S8. Number of shared and unique gene families of *M. atropurpurea*.........................11

Figure S9. Functional enrichment analysis of significant ($P < 0.05$) expanded gene family and contracted gene family in *M. atropurpurea*...12

Figure S10. Change rate of cross-validation (CV) error value of Admixture in K-values ranged from 1 to 12...13

Figure S11. Admixture of mulberry accessions based on different numbers of clusters (K = 2–12)...14

Figure S12. Relatedness estimation between kinship and population structure...15

Figure S13. Comparison of the mulberry accessions in the phylogenetic tree between accessions in this study and accessions published by Jiao et al. (2020).............16

Figure S14. Analysis of gene flow among different populations and geographic areas in mulberry..17

Figure S15. Estimates of the effective population size (Ne) for each subgroup of *M. alba* (MM and MA) and *M. atropurpurea* (Landrace1, Landrace2, and MECMA) using SMC++ 18

Figure S16. Demographic history of mulberry..19

Figure S17. Quantile-quantile plots for key agronomic traits GWAS analysis in the mulberry population...20

Figure S18. *MaERF110* sequence structure and alignment...21

Additional file 1: Tables... 22

Table S1. Comparison of sequence length of each chromosome between *M. atropurpurea* and *M. alba*.. 22

Table S2. Identification of genomic variation between *M. atropurpurea* and *M. alba*........... 23

Table S3. BUSCO and CEGMA evaluation of the genomic completeness of *M.atropurpurea*...24

Table S4. The statistics of annotated genes by different databases of *M. atropurpurea* 25

Table S5. The content of major TE subfamilies in the updated genome of *M. atropurpurea* (Female).................................26

Additional file 1: Figures

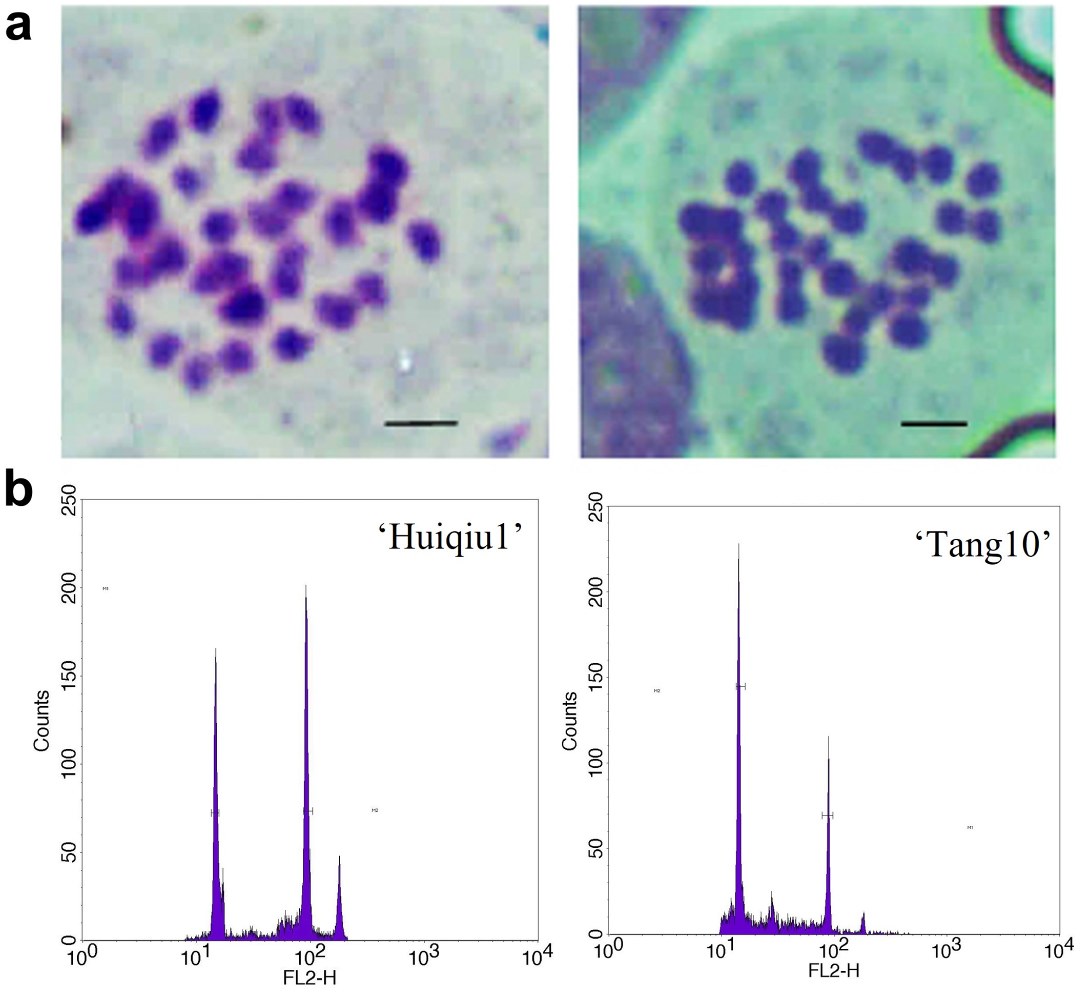

Figure S1. Analysis of chromosome ploidy of *M. atropurpurea*. (a) Chromosome number of *M. atropurpurea* "Tang 10" (2n = 28). Bars = 5 μm. (b) Estimation of genome ploidy for "Huiqiu1" and "Tang 10"using flow cytometry with Zea mays L. as an internal reference. The x-axis is the relative DNA quantity (Da). The y-axis is the number of counted cells.

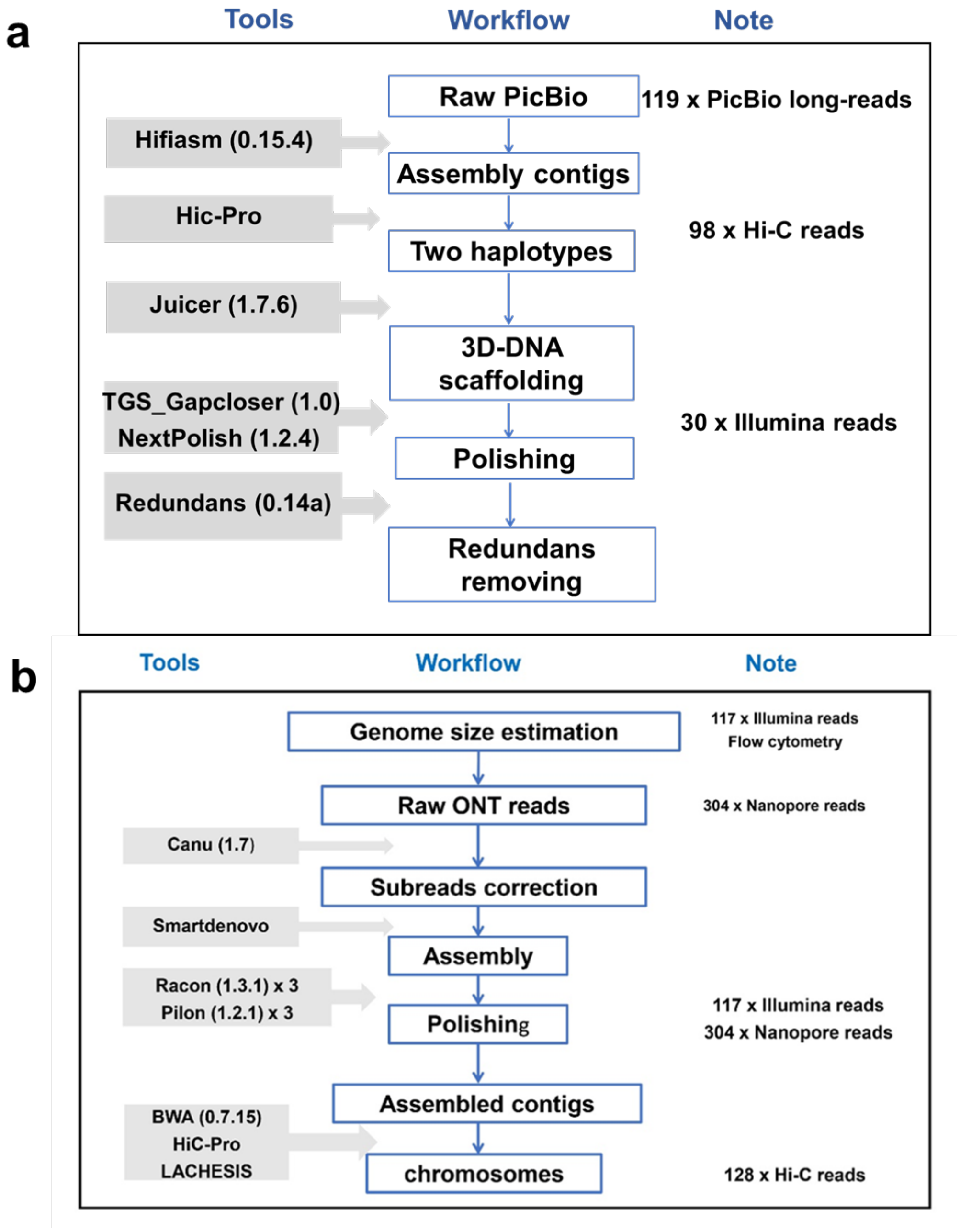

Figure S2. The flowchart of assembly for the *M. atropurpurea* genome. (a) Male cultivar 'Huiqiu1'. (b) Female cultivar 'Tang 10'.

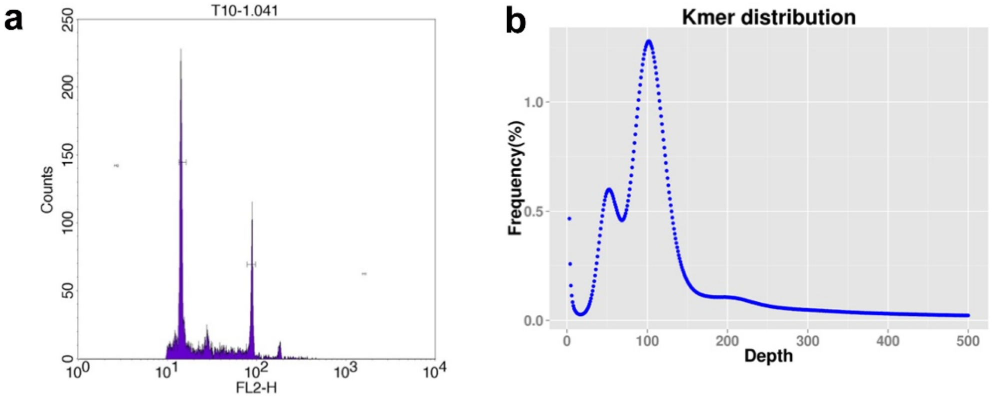

c

	Peak value of Reference plant	Peak value of *Morus atropurpurea*	Reference plant	Ratio	C value	Genome size (GB)
Morus atropurpurea	127.4	20.59	*Zea mays* L.	0.16	0.38	0.37
Morus atropurpurea	130.35	21.02	(Cultivar-B73)/	0.16	0.38	0.37
Morus atropurpurea	137.36	21.16	2.3 Gb	0.15	0.36	0.35
Mean						0.36

d

k-mer	Library inserted size	Main peak depth of k-mer	Number of filtered Kmer	Estimated genome Size	Heterozygote frequencies	Repetitive sequence
19	350 bp	103	31,953,256,456	308.07 Mb	0.0087	43.08%

Figure S3. The genome size estimate of *M. atropurpurea*. **(a)** Genome size of *M. atropurpurea* was measured by flow cytometry with *Zea mays* L. as an internal reference. The x-axis is the relative DNA quantity (Da). The y-axis is the number of counted cells. **(b-c)** The genome size was estimated by calculating the distribution of 19-mer frequency in the sequencing reads. The x-axis is the depth (X), and the y-axis is the proportion of sequences that represent the frequency at that depth divided by the total frequency of all depths. Overall, 35 Gb of data were retained for 19-mer analysis. The main distribution peak had approximately 71× coverage, and the genome size was estimated to be 308 Mb (Genome Size=K-mer number/Peak depth). The small peak at 1/2 of the main peak depth (approximately 36× coverage) shows the intermediate heterozygosity rate of the genome. The K-mer depth appears more than 2 times (approximately 142×coverage) at the corresponding depth of the main peak, representing repetitive sequences; that is, the K-mer sequence with depth greater than 142 is a repetitive sequence. **(d)** Estimate of mulberry genome size based on 19 K-mer statistics.

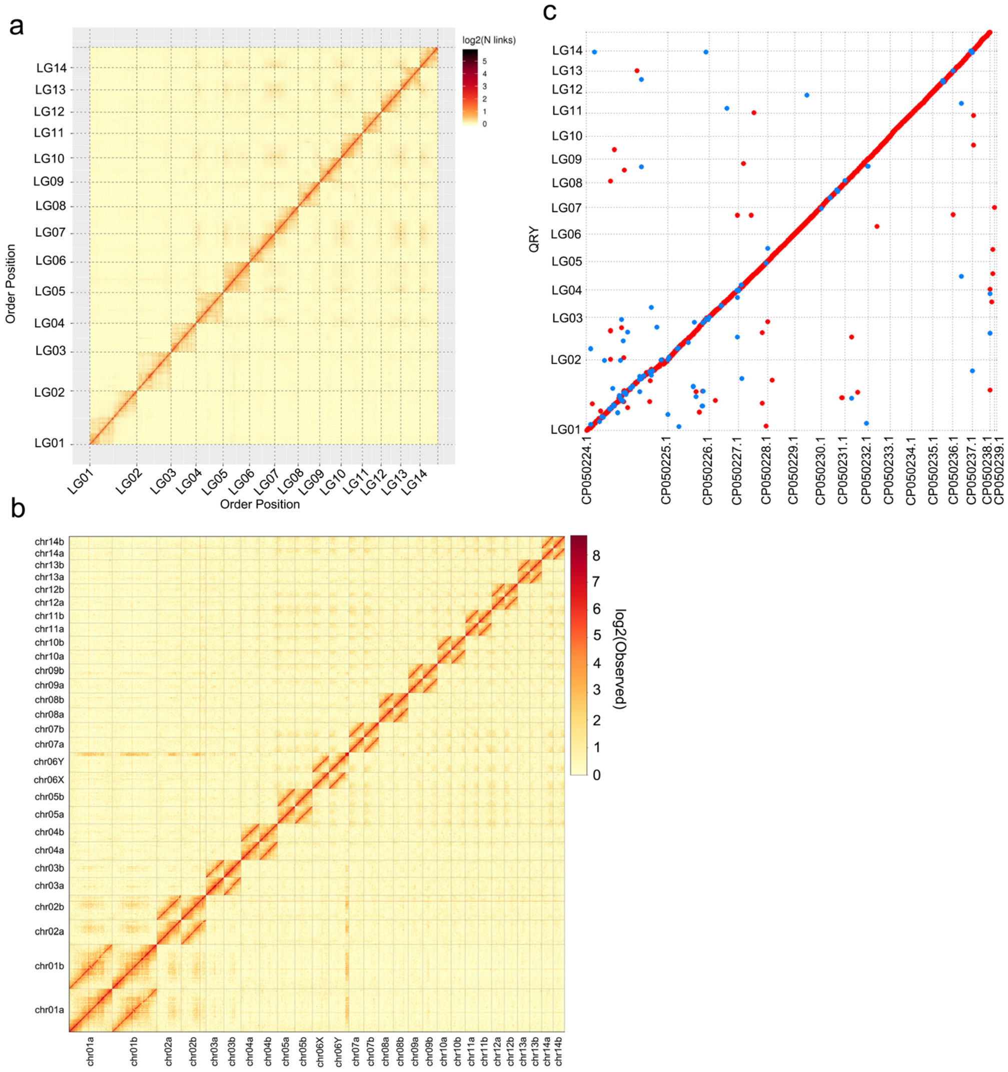

Figure S4. Collinearity analysis of *M. atropurpurea*. (a-b) Validation of Hi-C-assisted pseudochromosome assembly by calculating the thermal interaction correlation for 'Tang 10' and 'huiqiu1'. The coordinates of the dots represent the physical locations (x-axis) and map locations of the markers (y-axis). (c) Collinearity analysis of *M. atropurpurea* with *M. alba*. x-axis, *M. alba*; y-axis, *M. atropurpurea*.

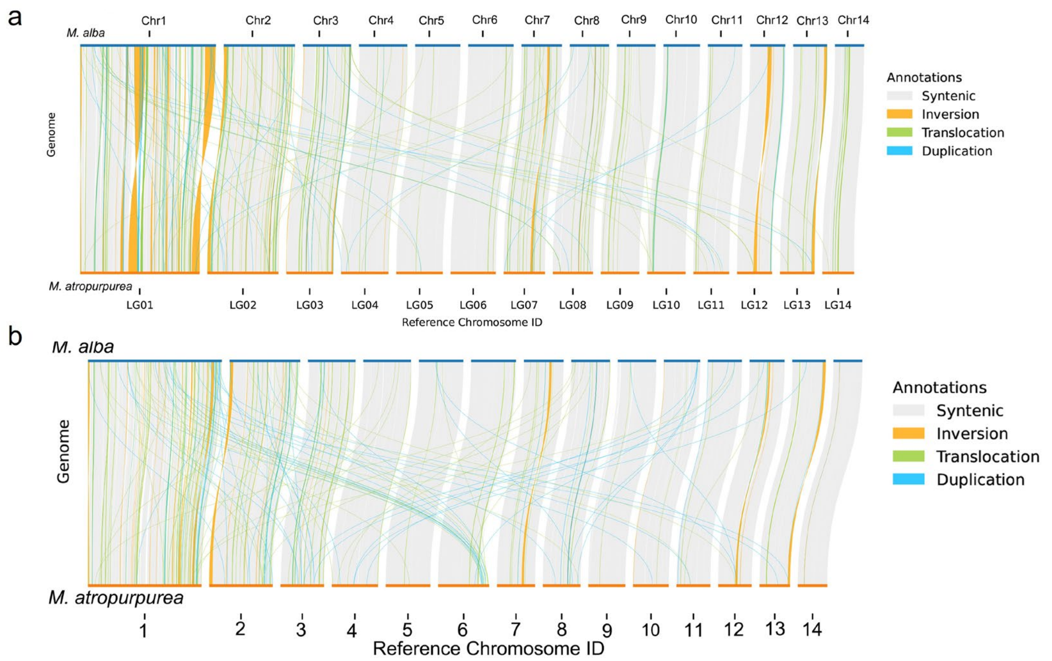

Figure S5. Chromosome synteny and structural variation between *M. atropurpurea* and *M. alba*. (a) Female cultivar 'Tang 10'. (b) Male cultivar 'Huiqiu1'.

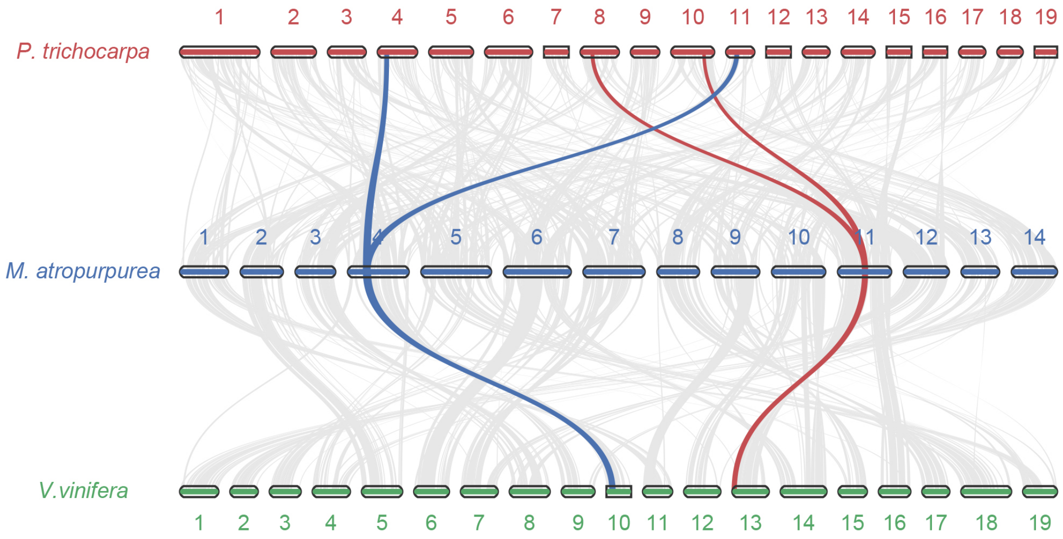

Figure S6. Schematic representation of syntenies among *M. atropurpurea*, *Populus trichocarpa* and grape genomes. Each line represents a syntenic region. Red lines highlight the one-to-one syntenic relationships between *M. atropurpurea* and grape or poplar.

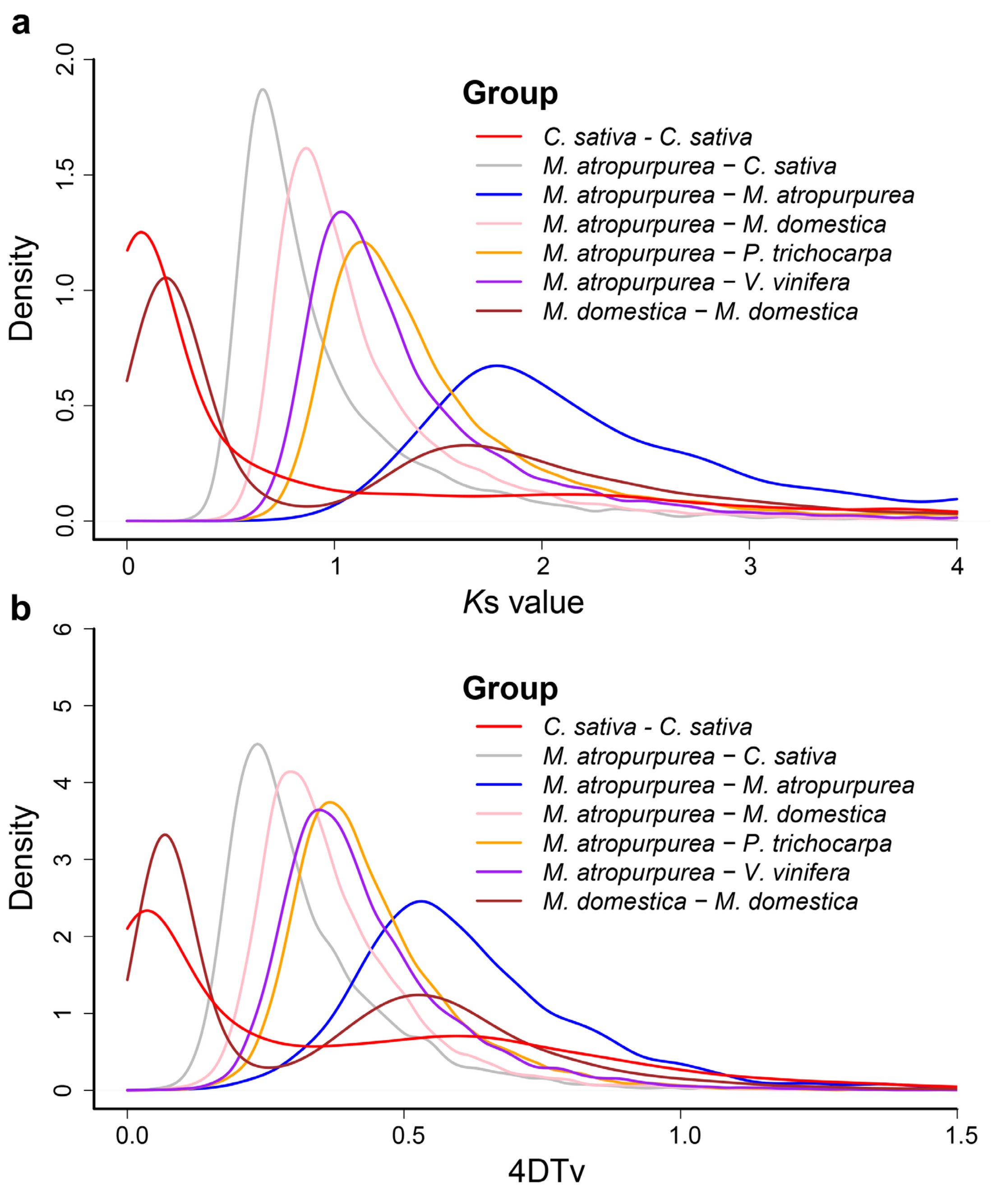

Figure S7. Density distribution of **(a)** 4DTv (fourfold synonymous third-codon transversion) and **(b)** *K*s for paralogous genes in *M. atropurpurea*. The color code of the curves is shown in the inset. The male plant was used for analysis.

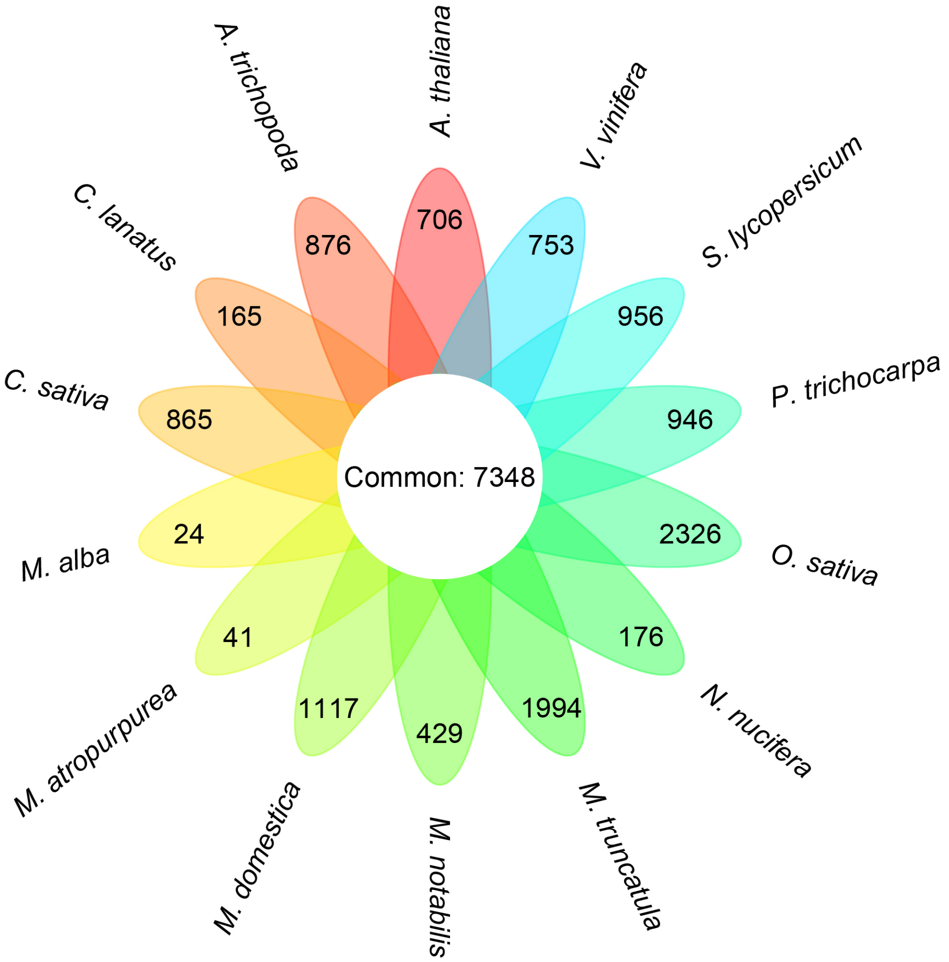

Figure S8. Number of shared and unique gene families of *M. atropurpurea*. Protein-coding genes from 14 species, including *Arabidopsis thaliana*, *Oryza sativa*, *Populus trichocarpa* (v3.0), *Solanum lycopersicum*, *Medicago truncatula*, *Vitis vinifera*, *Malus domestica*, *Nelumbo nucifera*, *Amborella trichopoda*, *Citrullus lanatus*, *Cannabis sativa*, *Morus notabilis*, *M. alba*, and *M. atropurpurea*. The male plant of *M. atropurpurea* was used for analysis.

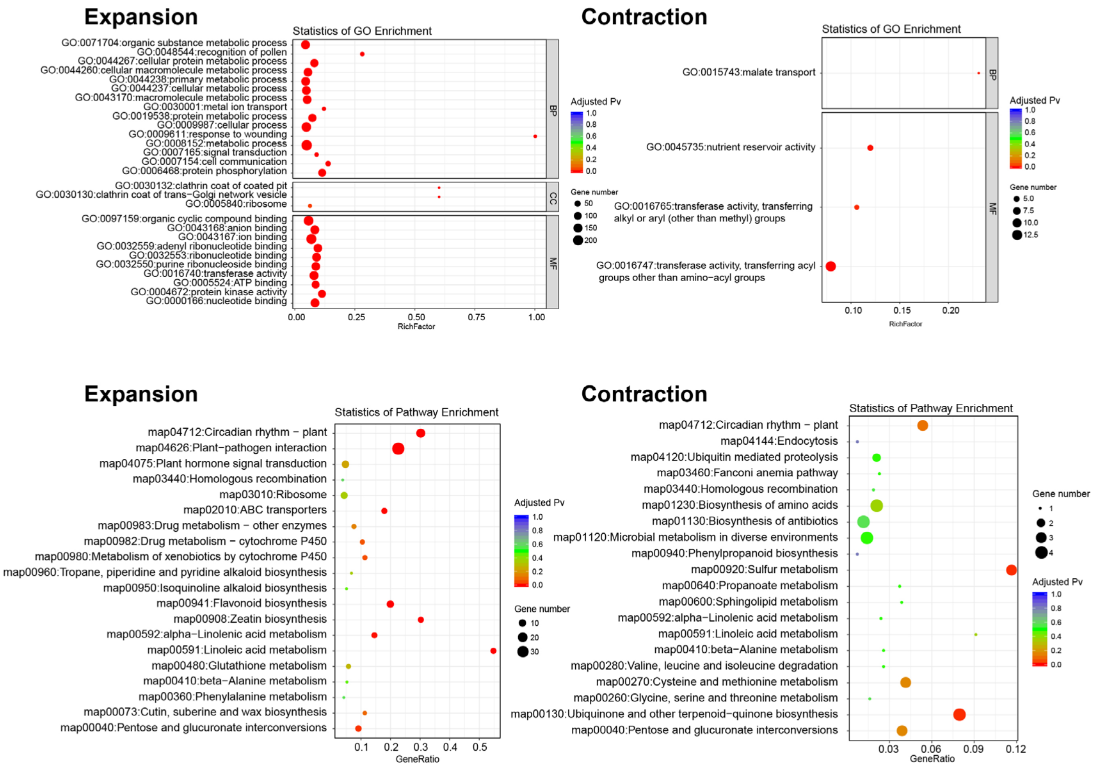

Figure S9. Functional enrichment analysis of significant (P<0.05) expanded gene family and contracted gene family in *M. atropurpurea*. **(a-b)** Gene Ontology (GO) functional analysis of **(a)** expanded gene families and **(b)** contracted gene families. **(c-d)** Kyoto Encyclopedia of Genes and Genomes (KEGG) enrichment analysis of **(c)** expanded gene families and **(d)** contracted gene families. The male plant was used for analysis.

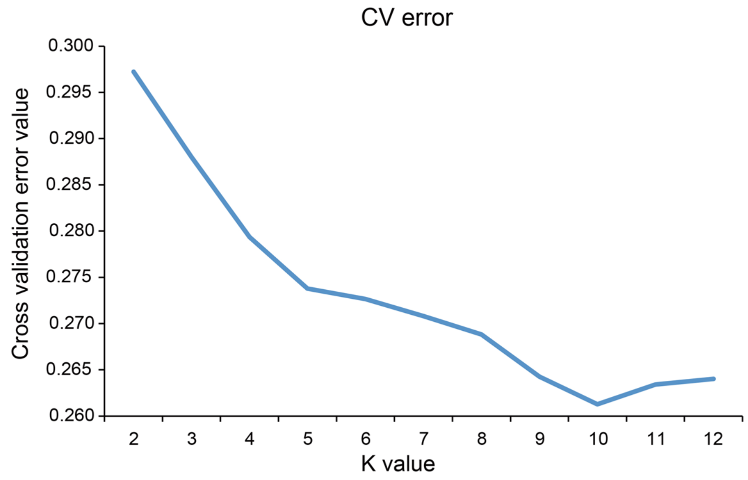

Figure S10. Change rate of cross-validation (CV) error value of admixture in K-values ranged from 1 to 12.

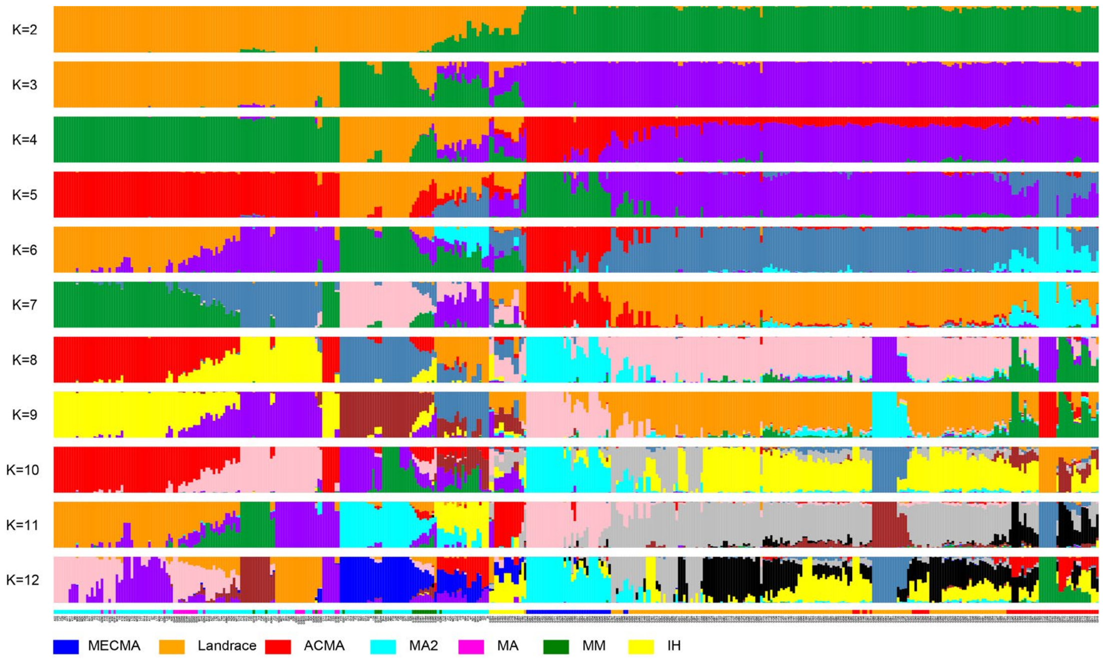

Figure S11. Admixture of mulberry accessions based on different numbers of clusters (K = 2–5). Population structure at K = 1–12 in which each individual is represented by a vertical color-coded column, inferred which subgroup the mulberry accession belongs to and the proportion of genetic components from ancestral populations.

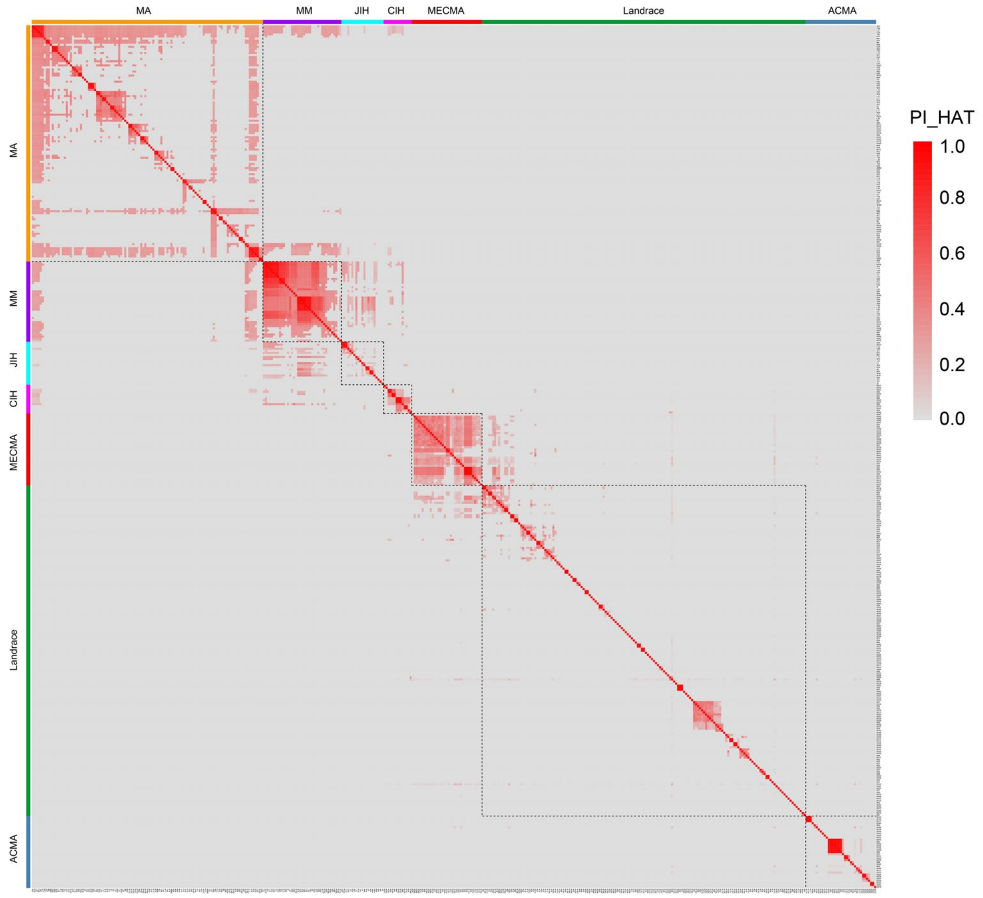

Figure S12. Relatedness estimation between kinship and population structure. PI_HAT indicates Proportion of Identity By Descent (IBD).

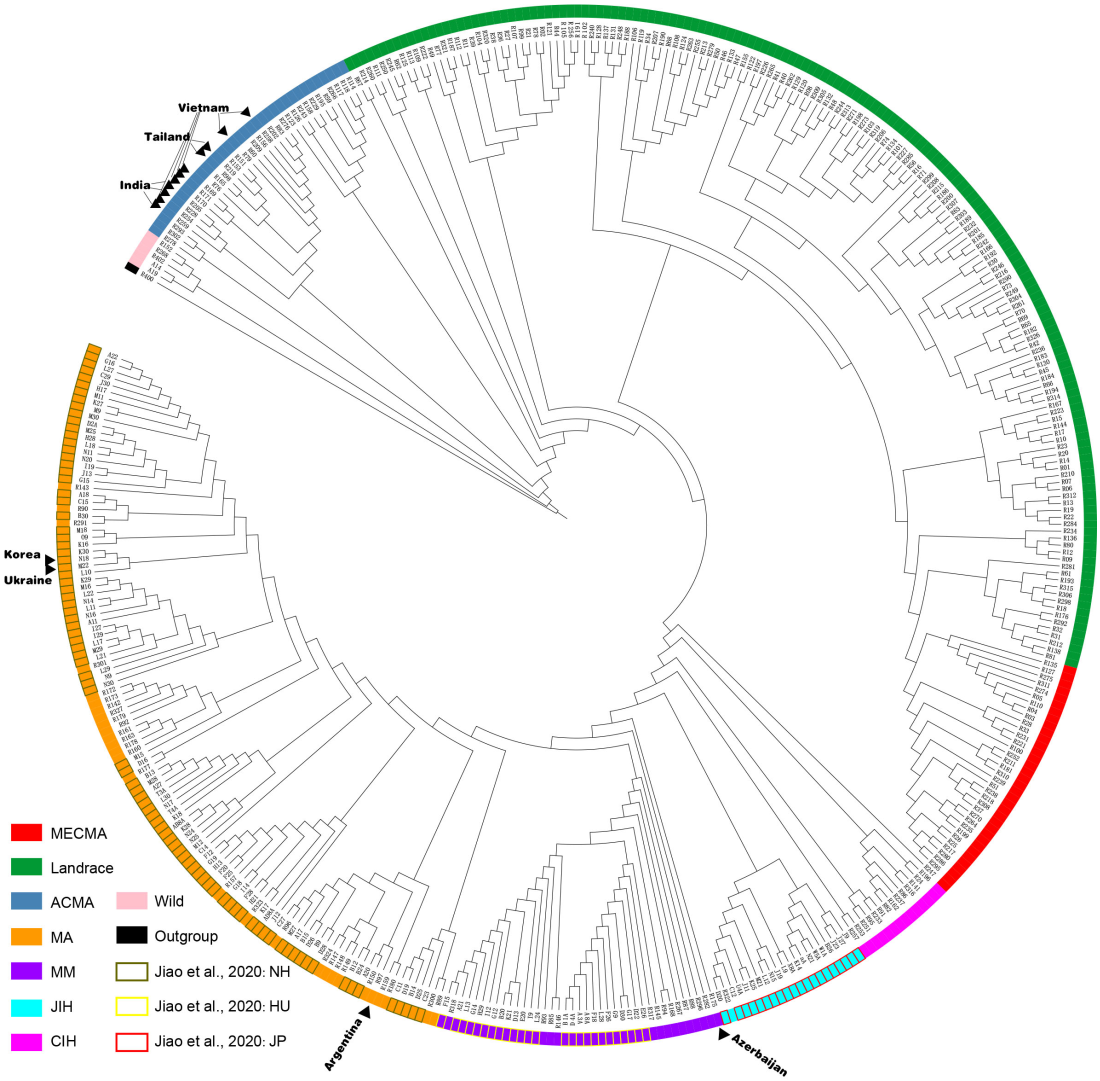

Figure S13. Comparison of the mulberry accessions in the phylogenetic tree between accessions in this study and accessions published by Jiao et al (2020).

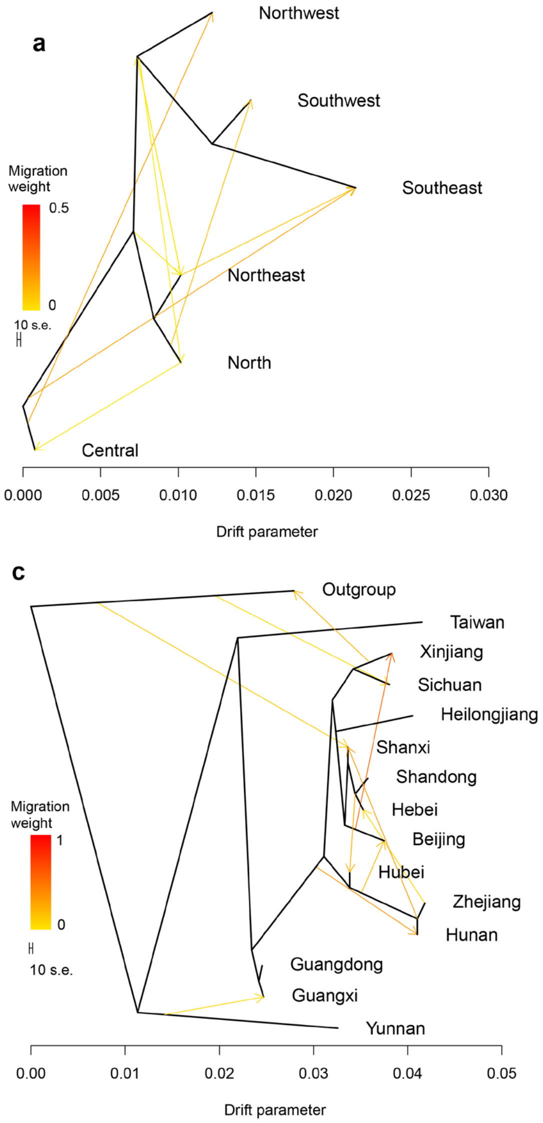

Figure S14. Analysis of gene flow among different populations and geographic areas in mulberry. **(a)** Inferred population splits and migrations among different geographic areas of China and **(b)** different genetic population of mulberry accessions from the TreeMix analysis. The orange line shows the potential migration events among groups. *M. atropurpurea* (including Landrace1, Landrace 2, and MECMA (elite cultivars) groups), CIH (Interspecific hybrid from China), JIH (Interspecific hybrid from Japan), MA (*M. alba*), and MM (*M. multicaulis*). **(c)** Population splits and migrations among accessions from different provinces of China. The arrows correspond to the direction of gene flow.

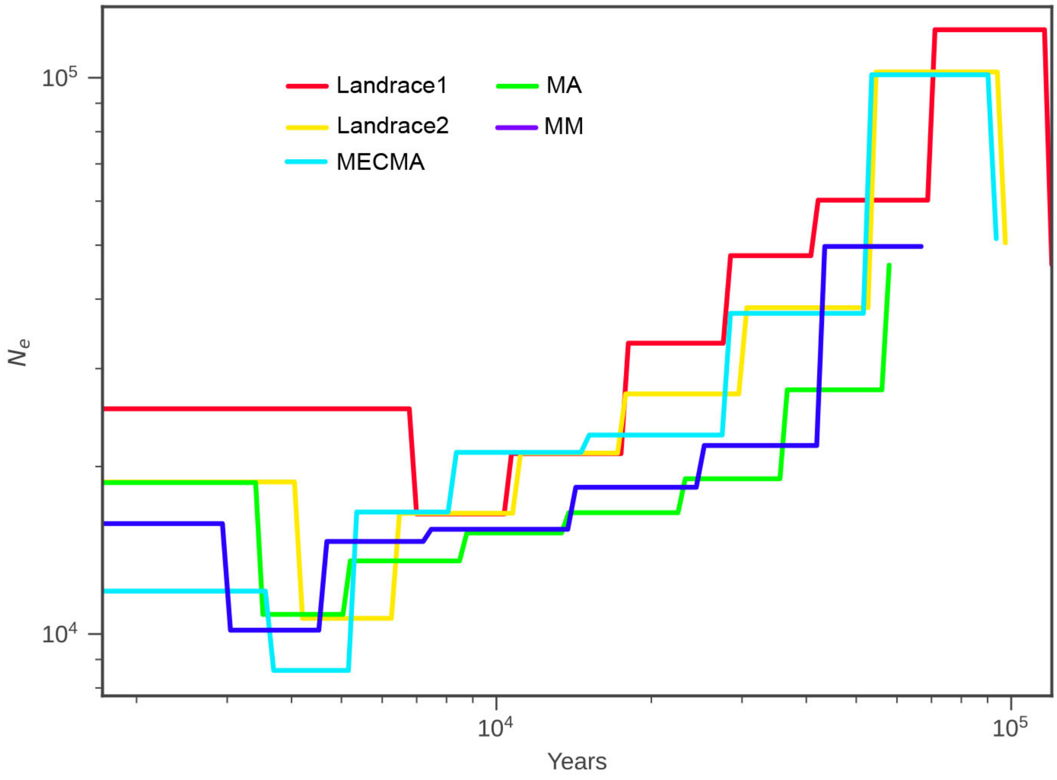

Figure S15. Estimates of the effective population size (Ne) for each subgroup of *M. alba* (MM and MA) and *M. atropurpurea* (Landrace1, Landrace2, and MECMA) using SMC++. We set the generation time (g) as 1 years and the mutation rate (μ) as 7e × 10^{-09} per site per generation.

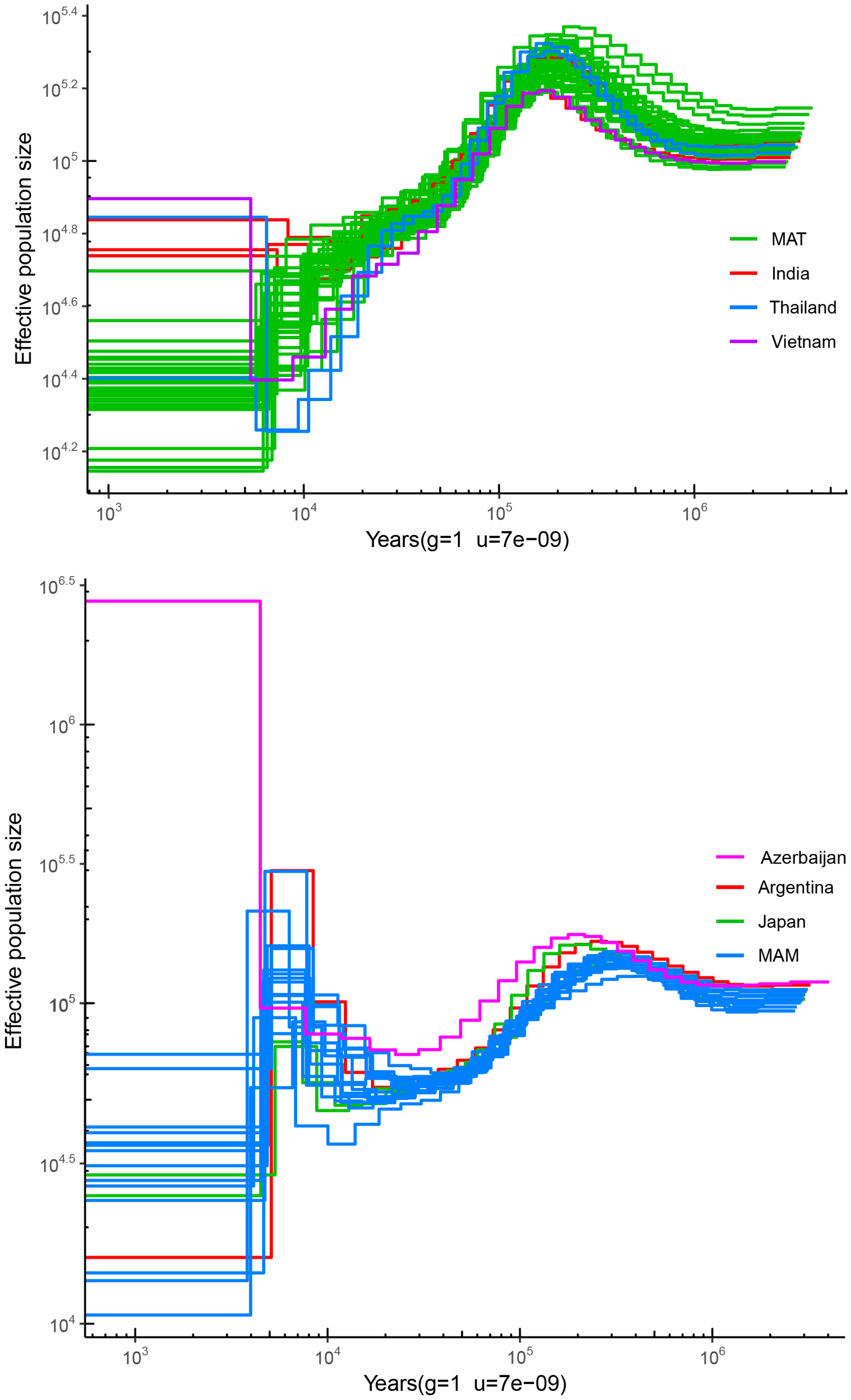

Figure S16. Demographic history of mulberry. **(a)** Comparison of effective population size change over time of mulberry from southeast Asian countries with MAT. MAT, *M. atropurpurea*. **(b)** Comparison of effective population size change over time of mulberry from Japan and Argentina with MAM. MAM, *M. alba* (MA) and *M. multicaulis* (MM).

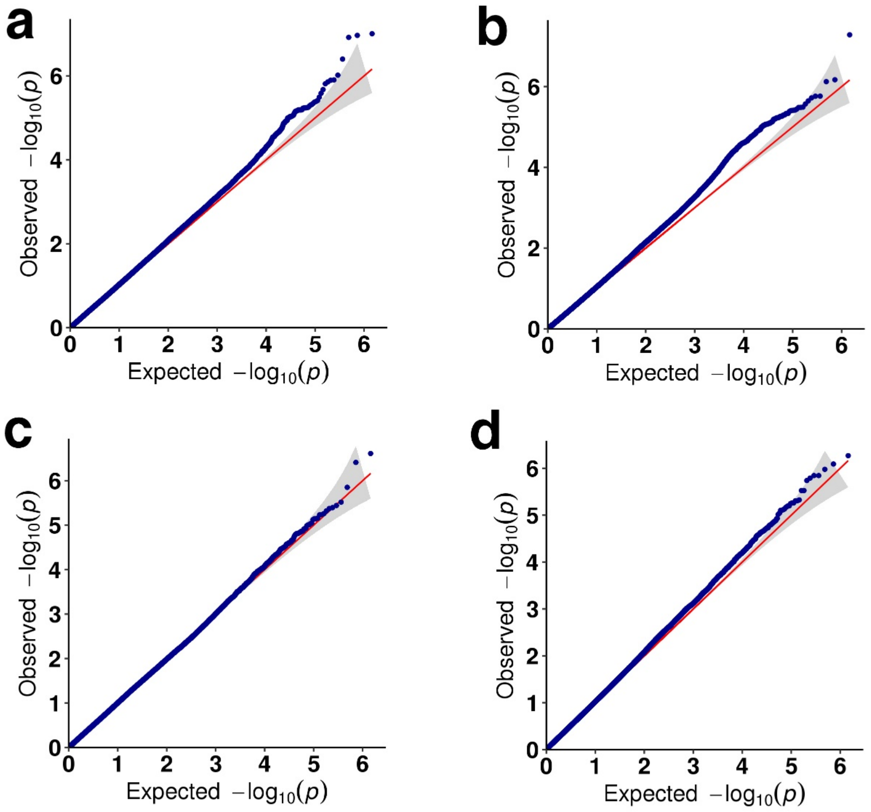

Figure S17 Quantile-quantile plots for key agronomic traits GWAS analysis in the mulberry population. The horizontal axis shows $-\log_{10}$ transformed expected *P* values, and the vertical axis indicates $-\log_{10}$ transformed observed *P* values. **(a)** Leaf size. **(b)** Leaf weight. **(c)** Flowering time. **(d)** Sex.

a

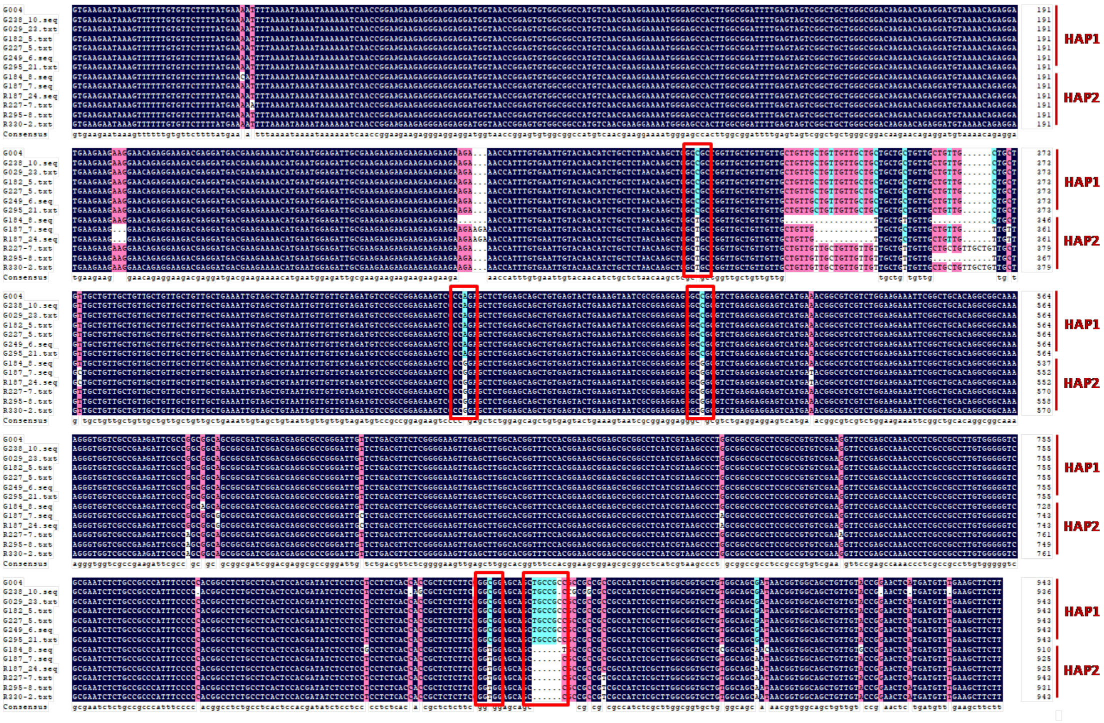

b

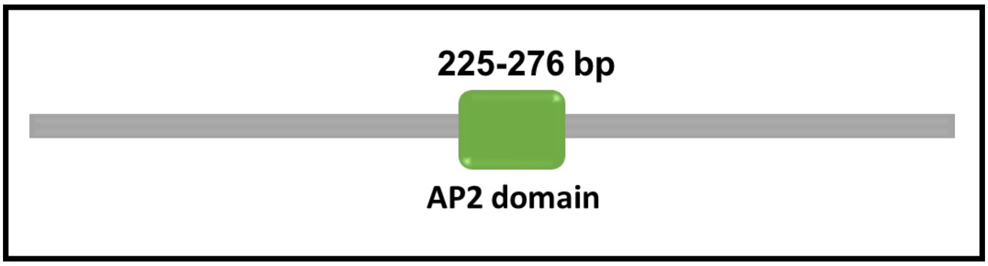

Figure S18. *MaERF11*0 sequence structure and alignment. **(a)** Alignment of the deduced amino acid sequence of *MaERF11*0 in the HAP1 and HAP2 association. The red columns indicate the candidate SNPs and indels associated with flowering time. **(b)** Identification of the AP2 domain in *MaERF11*0.

Additional file 1: Tables

Table S1. Comparison of sequence length of each chromosome between *M. atropurpurea* and *M. alba*.

Chromosome	*M. atropurpurea*			*M. alba*
	Male haplotype A	Male haplotype B	Female	
Chr1	55,087,750	53,033,940	57,088,720	64,903,500
Chr2	30,682,697	30,161,907	33,967,831	34,145,583
Chr3	21,063,469	22,186,303	22,427,243	23,008,319
Chr4	22,365,995	22,876,273	22,703,086	23,174,727
Chr5	21,588,871	21,559,954	22,236,475	21,537,881
Chr6	20,471,674	24,783,721	21,534,977	21,776,176
Chr7	18,350,245	18,466,990	19,800,448	19,270,414
Chr8	18,300,692	18,270,987	19,148,248	18,750,899
Chr9	17,977,237	17,846,368	18,426,848	18,524,754
Chr10	17,258,240	17,253,113	18,369,319	17,554,266
Chr11	16,342,456	16,350,467	17,079,887	16,501,500
Chr12	16,271,622	15,986,940	16,749,152	16,789,328
Chr13	14,815,440	15,041,847	16,524,587	16,207,330
Chr14	14,359,216	14,130,079	15,185,383	13,983,734
Total	304,935,604 (99.90%)	307,948,889 (99.18%)	321,242,204 (97.65%)	326,128,411 (94.15%)

Table S2. Identification of genomic variation between *M. atropurpurea* and *M. alba*.

Variation type	*M. atropurpurea* (Male) vs *M. alba*			*M. atropurpurea* (Female) vs *M. alba*		
	**Count**	**Length of *M. alba***	**Length of *M. atropurpurea***	**Count**	**Length of *M. alba***	**Length of *M. atropurpurea***
Syntenic regions	659	27,458,1114	27,517,507	614	268561605	275963540
Inversions	56	7,826,813	7,269,361	61	21907333	19968291
Translocations	541	5,765,452	5,796,385	435	10453344	10435396
Duplications (*M. alba*)	193	111,722	-	203	1628471	-
Duplications (*M. atropurpurea*)	505	-	1,862,324	318	-	2586114
Not aligned (*M. alba*)	1,243	3,784,4714	-	1035	29902189	-
Not aligned (*M. atropurpurea*)	1,471	-	21,745,586	1197	-	14996723
SNPs	1,575,194	1,575,194	1,575,194	1668662	1668662	1668662
Insertions	225,610	-	1,868,350	242682	-	1935445
Deletions	207,474	1,758,395	-	295037	1825315	-
Copygains	88	-	357,215	112	-	1663594
Copylosses	96	57,9256	-	102		-
Highly diverged region (HDR)	34,336	100,715,190	100,889,418	40724	107289917	111931174
Tandem repeats	25	2,952	3,136	22	1655	2039

Table S3. BUSCO and CEGMA evaluation of the genomic completeness of *M. atropurpurea*.

		Male haplotype A		Male haplotype B		Female	
		Number	Proportion (%)	Number	Proportion (%)	Number	Proportion (%)
BUSCO	Complete BUSCO (C)	1576	97.6	1572	97.4	1,557	96.47
	Complete and single-copy BUSCO (S)	1556	96.4	1552	96.2	1,524	94.42
	Complete and duplicated BUSCO (D)	20	1.2	20	1.2	33	2.04
	Fragmented BUSCO (F)	17	1.1	22	1.4	33	2.03
	Missing BUSCO (M)	21	1.3	20	1.2	24	1.49
	Total BUSCO groups	1614	100	1614	100	1614	100
CEGMA	Number of 458 CEG	430	93.89	421	91.92	452	98.69
	Number of 248 highly conserved CEGs	231	93.15	227	91.53	229	92.34

Table S4. The statistics of annotated genes by different databases of *M. atropurpurea*.

Database	Female haplotype A		Male haplotype B		Female	
	Annotated number	Percentage (%)	Annotated number	Percentage (%)	Annotated number	Percentage (%)
GO	10,863	51.50	10,859	51.95	12,739	49.62
KEGG	19,696	93.38	19,767	94.57	8,399	32.71
KOG	14,888	70.59	14,984	71.69	12,851	50.05
TrEMBL	20,670	98.00	20,744	99.25	25,340	98.7
nr	20,661	97.96	20,715	99.11	25,332	98.66
All	20,723	98.25	20,782	99.43	25,355	98.75

Table S5. The content of major TE subfamilies in the updated genome of *M. atropurpurea* (Female).

Type	Number	Length	Rate (%)
ClassI	430,745	148,954,742	45.28
ClassI/DIRS	10,029	6,686,421	2.03
ClassI/LARD	199,012	38,266,523	11.63
ClassI/LINE	7,292	1,618,951	0.49
ClassI/LTR/Copia	124,119	63,654,675	19.35
ClassI/LTR/Gypsy	84,021	48,018,200	14.6
ClassI/LTR/Unknown	3,567	834,760	0.25
ClassI/PLE	1,620	204,526	0.06
ClassI/SINE	4	218	0
ClassI/TRIM	1,017	2,209,648	0.67
ClassI/Unknown	64	3,901	0
ClassII	83,506	21,473,618	6.53
ClassII/Crypton	9	496	0
ClassII/Helitron	25,830	6,846,348	2.08
ClassII/Maverick	1,001	1,005,184	0.31
ClassII/TIR	49,639	13,451,902	4.09
ClassII/Unknown	7,027	626,204	0.19
PotentialHostGene	26,663	4,870,007	1.48
SSR	674	177,121	0.05
Unknown	47,266	9,143,601	2.78
**Total**	**588,854**	**173,159,115**	**52.63**

Table S6. The content of major TE subfamilies in the updated genome of *M. atropurpurea* (Male).

Order	Superfamily	Haplotype A			Haplotype B		
		Number	Length(bp)	Percent(%)	Number	Length(bp)	Percent(%)
LTR		150,122	86,967,845	14.11	145,616	82,241,890	13.34
	Copia	62,858	32,124,721	5.21	58,230	30,146,531	4.89
	Gypsy	54,253	35,884,433	5.82	56,564	34,377,194	5.58
	unknown	33,011	18,958,691	3.08	30,822	17,718,165	2.87
	pararetrovirus	32	33	9,503	0.00	32	8,579
LINE		505	348,593	0.06	518	340,587	0.06
DNA		252,143	53,819,198	8.73	255,572	53,854,566	8.74
	DTA	14,024	6,229,405	1.01	13,633	6,124,120	0.99
	DTC	32,861	7,861,027	1.28	32,455	7,699,610	1.25
	DTH	34,789	5,574,361	0.90	39,967	6,159,785	1.00
	DTM	29,750	6,147,884	1.00	29,508	6,186,091	1.00
	DTT	4,956	1,166,368	0.19	4,984	1,173,458	0.19
	Helitron	135,763	26,840,153	4.35	135,025	26,511,502	4.30
TIR		1,262	805,982	0.13	1,264	809,522	0.13
	EnSpm_CACTA	174	114,890	0.02	179	127,196	0.02
	MuDR_Mutator	713	543,344	0.09	709	531,558	0.09
	PIF_Harbinger	309	123,468	0.02	318	128,313	0.02
	hAT	66	24,280	0.00	58	22,455	0.00
MITE		10,643	2,275,752	0.37	10,401	2,263,345	0.37
	DTA	2,043	758,487	0.12	2,024	761,985	0.12
	DTC	279	78,316	0.01	288	79,889	0.01
	DTH	6,460	1,058,727	0.17	6,405	1,058,429	0.17
	DTM	1,860	380,051	0.06	1,683	362,871	0.06
	DTT	1	171	0.00	1	171	0.00
Unknown		54,460	14,138,624	2.29	58,372	14,218,385	2.31
Simple_repeat		210,885	211,289	8,542,040	1.39	210,885	210,885
Low_complexity		32,669	32,644	1,742,459	0.28	32,669	32,669
**Ttotal**		**713,101**	**168,649,996**	**55.24**	**715,329**	**163,850,550**	**52.77**

Table S7. Gene prediction of the *M. atropurpurea* genome.

Parameter	Male		Female
	Haplotype A	Haplotype B	
Total number of gene	21,092	20,901	25,675
Gene Length (bp)	80,309,411	78,935,821	106,770,156
Average gene length (bp)	4,524	4,548	4,159
Number of rRNA gene	201	198	119
Number of tRNA gene	425	424	458
Number of miRNA gene	94	97	69

Table S8. Statistics of calling variations in all accessions.

Parameter	SNP	Indel	Affected genes
Total	2359117	934187	-
Genic	1310964	494423	-
Exonic	215622	16134	-
Frameshift	-	10175	5876
Indels that change stop codons	-	519	498
Indels that change start codons	-	113	113
Codon insertion or deletion	-	5327	4242
Non-synonymous	115791	-	19796
Synonymous	91093	-	18590
SNPs that change stop codons	7299	-	5986
SNPs that change start codons	1439	-	1283
Intronic	439350	176710	-
Upstream	287177	134350	-
Downstream	279171	125408	-
3’ or 5’ UTR PRIME	75859	37466	-
Intergenic	1048153	439764	-

Table S9. Analysis of Patterson's *D* among different mulberry subpopulations.

P1	P2	P3	Dstatistic	Z-score	p-value	f4-ratio	BBAA	ABBA	BABA
Landrace1	Landrace2	JIH	0.033	6.637	3.2E-11	0.145	24614.2	20882.4	19566.3
Landrace1	JIH	MA	0.041	4.556	5.2E-06	0.152	19478.7	23619.9	21765.2
Landrace1	MECMA	JIH	0.040	4.249	2.1E-05	0.174	23771.6	20743.4	19160.4
JIH	MM	Landrace1	0.086	7.149	8.7E-13	0.315	27174.3	20491.2	17235.5
MA	MM	Landrace1	0.027	3.738	0.00019	0.119	26920.2	18322.4	17365.8
Landrace2	CIH	JIH	0.034	3.290	0.001	0.175	23270	20925.5	19561.9
CIH	MA	JIH	0.036	4.023	5.8E-05	0.228	22971.7	20772.5	19313.7
MECMA	CIH	JIH	0.028	3.087	0.00202	0.146	23022.6	20217.9	19120.9
CIH	MA	MM	0.054	7.256	4E-13	0.168	17128.1	22626.7	20306.1
Landrace2	JIH	MM	0.093	9.713	0	0.244	16847.7	25471.1	21130.3
JIH	MA	MM	0.042	3.899	9.6E-05	0.142	16190.8	23885.6	21981.5
MECMA	JIH	MM	0.085	8.216	2.1E-16	0.224	16406.1	24762.9	20885
MA	MM	Landrace2	0.026	3.526	0.00042	0.215	24813.9	18558.9	17635.4

Table S10. Identification of candidate genes associated with leaf size.

GeneID	Gene Start	Gene End	Gene annotation
EVM0019929.1	9723436	9726990	putative endo-1,4-beta-xylanase [Morus notabilis]
EVM0015244.1	9732244	9736802	Endo-1,4-beta-xylanase C [Morus notabilis]
EVM0014670.1	9741287	9744357	Adenylyl-sulfate kinase 1 [Morus notabilis]
EVM0002233.1	9744929	9746892	Endo-1,4-beta-xylanase C [Morus notabilis]
EVM0003088.1	9747524	9748579	Endo-1,4-beta-xylanase C [Morus notabilis]
EVM0022813.1	9757440	9762284	Endo-1,4-beta-xylanase C [Morus notabilis]
EVM0016984.1	9773146	9776875	Adenylyl-sulfate kinase 1 [Morus notabilis]
EVM0004381.1	9784636	9790548	DNA polymerase kappa [Morus notabilis]
EVM0015468.1	9791244	9792165	hypothetical protein [Morus notabilis]
EVM0021942.1	9793183	9795902	Mitochondrial outer membrane protein porin 2 [Morus notabilis]
EVM0015901.1	9797027	9799596	Mitochondrial outer membrane protein porin 2 [Morus notabilis]
EVM0008101.1	9805329	9808205	hypothetical protein [Morus notabilis]
EVM0003006.1	9808640	9811838	Ribosomal RNA large subunit methyltransferase N 1 [Morus notabilis]
EVM0011993.1	9812558	9812875	hypothetical protein [Morus notabilis]
EVM0001601.1	9815785	9818825	hypothetical protein [Morus notabilis]
EVM0000101.1	9818936	9820060	hypothetical protein [Morus notabilis]
EVM0015335.1	9820766	9825829	putative beta-D-xylosidase

Table S11. Significant SNPs associated with mulberry leaf size (-log10(P) > 8).

Chr.	SNP position	logP	Ref	Alt	SNP annotation	SNP associated gene
7	9,749,773	8.45	T	C	Upstream	*EVM0003088.1*
7	9,749,870	9.71	C	A	Upstream	*EVM0003088.1*
7	9,787,152	9.33	G	A	Intron	*EVM0004381.1*
7	9,789,892	10.57	G	A	Intron	*EVM0004381.1*
7	9,789,951	10.57	G	A	Intron	*EVM0004381.1*
7	9,790,230	10.63	A	G	Synonymous	*EVM0004381.1*
7	9,790,325	10.69	G	C	Non_synonymous	*EVM0004381.1*
7	9,790,412	10.31	A	G	Intron	*EVM0004381.1*
7	9,790,435	10.38	A	G	Non_synonymous	*EVM0004381.1*
7	9,790,452	10.38	G	C	Synonymous	*EVM0004381.1*
7	9,790,486	10.79	C	A	Non_synonymous	*EVM0004381.1*
7	9,791,054	10.93	C	A	Upstream	*EVM0015468.1*
7	9,791,817	9.70	C	T	Intron	*EVM0015468.1*

Table S12. SNP analysis associated with flowering time (-log10(*P*) ⩾ 8).

Chr.	SNP position	-log10(*P*)	Ref	Alt	SNP annotation	SNP associated gene
5	20,726,465	10.12	G	T	downstream	*EVM0001723.1*
5	20,727,787	9.67	C	A	downstream	*EVM0001723.1*
5	20,732,086	9.16	C	A	intergenic	-
5	20,732,635	15.41	G	A	intergenic	-
5	20,734,419	19.31	G	C	intergenic	-
5	20,734,430	14.60	A	G	intergenic	-
5	20,734,846	15.41	A	G	intergenic	-
5	20,735,035	9.70	C	A	intergenic	-
5	20,736,467	16.92	T	G	intergenic	-
5	20,736,646	11.97	C	G	intergenic	-
5	20,736,655	8.99	A	G	intergenic	-
5	20,737,139	8.33	G	A	intergenic	-
5	20,738,722	23.15	A	T	intergenic	-
5	20,738,811	10.69	T	A	intergenic	-
5	20,739,302	8.65	A	G	intergenic	-
5	20,739,810	14.87	C	T	intergenic	-
5	20,739,876	11.61	T	C	intergenic	-
5	20,740,254	9.30	A	G	intergenic	-
5	20,740,411	13.05	A	G	intergenic	-
5	20,740,851	11.96	C	T	intergenic	-
5	20,740,872	14.66	C	T	intergenic	-
5	20,741,308	24.88	G	A	intergenic	-
5	20,741,672	14.69	G	T	intergenic	-
5	20,742,102	12.10	C	T	intergenic	-
5	20,742,130	9.44	A	T	intergenic	-
5	20,742,586	12.25	A	C	utr_3_prime	*EVM0010692.1*
5	20,742,949	13.63	A	G	utr_3_prime	*EVM0010692.1*
5	20,742,970	8.77	G	T	utr_3_prime	*EVM0010692.1*
5	20,743,409	9.95	C	T	non_synonymous	*EVM0010692.1*
5	20,743,547	11.29	A	G	non_synonymous	*EVM0010692.1*
5	20,743,595	9.87	C	G	non_synonymous	*EVM0010692.1*
5	20,743,934	13.63	C	T	non_synonymous	*EVM0010692.1*
5	20,744,464	12.34	T	A	intron	*EVM0010692.1*
5	20,744,547	13.16	G	T	intron	*EVM0010692.1*
5	20,745,864	13.34	C	T	intergenic	-
5	20,746,296	10.36	A	G	intergenic	-
5	20,747,118	12.01	G	A	intergenic	-
5	20,747,209	8.52	T	A	intergenic	-
5	20,747,273	10.75	A	T	intergenic	-
5	20,747,290	10.53	A	G	intergenic	-
5	20,747,382	8.32	C	T	intergenic	-
5	20,748,668	11.31	A	G	intergenic	-
5	20,749,186	11.70	T	C	intergenic	-
5	20,749,891	27.96	C	T	intergenic	-
5	20,750,604	26.00	T	C	intergenic	-
5	20,750,622	26.00	T	C	intergenic	-
5	20,750,750	21.17	G	A	intergenic	-
5	20,751,537	12.18	A	C	intergenic	-
5	20,751,587	26.00	T	A	intergenic	-
5	20,751,620	11.30	T	A	intergenic	-
5	20,752,074	26.18	A	C	intergenic	-
5	20,752,112	23.58	A	C	intergenic	-
5	20,752,510	15.75	A	C	intergenic	-
5	20,752,524	10.18	A	T	intergenic	-
5	20,754,036	8.35	T	C	intergenic	-
5	20,754,551	24.57	T	C	intergenic	-
5	20,754,742	24.44	G	T	intergenic	-
5	20,754,929	11.96	T	C	intergenic	-
5	20,755,339	19.51	A	G	intergenic	-
5	20,760,024	8.17	C	T	intergenic	-
5	20,764,797	8.16	C	T	non_synonymous	*EVM0000848.1*
5	20,765,551	8.07	T	G	intron	*EVM0000848.1*
5	20,765,688	8.16	A	T	synonymous	*EVM0000848.1*
5	20,765,794	8.16	T	C	intron	*EVM0000848.1*
5	20,766,968	9.61	T	C	utr_3_prime	*EVM0000848.1*
5	20,771,594	11.54	T	A	intron	*EVM0014750.1*

Table S13. Identification of candidate genes associated with flowering time.

Genes located in candidate region	Chr.	Gene start	Gene end	Function	FPKM value			
					G029 (EF[1])	G227 (EF)	G295 (LF[2])	G330 (LF)
EVM0007713.1	5	20,693,724	20,696,517	Peroxidase 12	0.10	0.30	0.17	0.00
EVM0001723.1	5	20,722,911	20,726,378	Putative invertase inhibitor	0.15	0.00	0.00	0.00
EVM0010692.1	5	20,742,415	20,745,474	Ethylene-responsive transcription factor	1.24	2.61	9.35	8.61
EVM0000848.1	5	20,761,824	20,767,227	Uncharacterized protein	15.41	19.71	39.56	27.39
EVM0014750.1	5	20,768,074	20,772,578	Methenyltetrahydrofolate synthase domain-containing protein	11.00	11.55	14.84	10.61

*[1] EF, early flower; [2] LF, late flower.

Table S14. Indels located at *ERF110*.

Chr.	Indel position	Ref	Alt	Indel annotation	Indel associated gene
5	20,742,418	TG	T	UTR_3_PRIME	*EVM0010692*
5	20,742,509	A	AC	UTR_3_PRIME	*EVM0010692*
5	20,742,562	CAAA	C	UTR_3_PRIME	*EVM0010692*
5	20,742,737	A	ATGCT,ATT	UTR_3_PRIME	*EVM0010692*
5	20,742,792	ATT	A	UTR_3_PRIME	*EVM0010692*
5	20,742,807	TTTG	T	UTR_3_PRIME	*EVM0010692*
5	20,742,861	TA	T	UTR_3_PRIME	*EVM0010692*
5	20,742,904	CGATAG	C	UTR_3_PRIME	*EVM0010692*
5	20,742,921	T	TAAA	UTR_3_PRIME	*EVM0010692*
5	20,742,934	TGA	T	UTR_3_PRIME	*EVM0010692*
5	20,742,959	AAT	A	UTR_3_PRIME	*EVM0010692*
5	20,743,012	TA	T	UTR_3_PRIME	*EVM0010692*
5	20,743,024	AAC	A	UTR_3_PRIME	*EVM0010692*
5	20,743,040	ATTTTTT	A,ATT,ATTTT,ATTTTT ,ATTTTTTT	UTR_3_PRIME	*EVM0010692*
5	20,743,063	T	TA,TAA	UTR_3_PRIME	*EVM0010692*
5	20,743,078	A	ATAAGAGGAAAAAAAAACTATGGTGTCCTGATAGG,ATAAGGGGAAAAAAAAACTATGCTGTCCTGATAGG,ATAAGGGAAAAAAAAAAAACTATGGTGTCCTGATAGG,ATAAGGGGAAAAAAAAAATTCTGCTGTCCTGATAGG,ATAAGGGGAAAAAAAAACTATGGTGTCCTGATAGG	UTR_3_PRIME	*EVM0010692*
5	20,743,107	GTT	G	UTR_3_PRIME	*EVM0010692*
5	20,743,286	TGAA	T	CODON_DELETION	*EVM0010692*
5	20,743,343	CGAAGAAGAA	CGAAGAAGAAGAA, CGAAGAA,CGAA,C	CODON_INSERTION	*EVM0010692*
5	20,743,484	CTGT	C	CODON_DELETION	*EVM0010692*
5	20,743,943	TGCCGCC	C	CODON_DELETION	*EVM0010692*
5	20,744,081	G	GA,GAA	INTRON	*EVM0010692*
5	20,744,111	C	CA	INTRON	*EVM0010692*
5	20,744,176	CA	C,CAA,CAAAAAAA, CAAA,CAAAAAAAA	INTRON	*EVM0010692*
5	20,744,231	A	ACT	INTRON	*EVM0010692*
5	20,744,458	A	AC	INTRON	*EVM0010692*
5	20,744,644	TTTC	T	CODON_DELETION	*EVM0010692*
5	20,745,177	GTCTC	G	UTR_5_PRIME	*EVM0010692*
5	20,745,208	T	TAA	UTR_5_PRIME	*EVM0010692*
5	20,745,311	A	AT,AAATAT	UTR_5_PRIME	*EVM0010692*
5	20,745,364	TA	T	UTR_5_PRIME	*EVM0010692*
5	20,745,458	T	TG	UTR_5_PRIME	*EVM0010692*

Table S15. Identification of sex-associated candidate genes specifically located in SDR-Y.

NO.	Genes located in candidate region	Chr.	Gene start	Gene end	Gene annotation
1	Moatr06Yg0184600.3	6	19922095	19924290	Hypothetical protein
2	Moatr06Yg0184700.1	6	19925684	19927821	Pentatricopeptide repeat-containing protein, mitochondrial
3	Moatr06Yg0184800.1	6	20051620	20055570	Gag/pol protein
4	Moatr06Yg0184900.1	6	20354443	20363517	Gag/pol protein
5	Moatr06Yg0187500.1		20729885	20733217	hypothetical protein
6	Moatr06Yg0187600.1		20752083	20756033	Gag/pol protein
7	Moatr06Yg0187800.1	6	20957728	20961678	Gag/pol protein
8	Moatr06Yg0188000.1	6	21014323	21018595	Hypothetical protein
9	Moatr06Yg0188100.1	6	21037154	21037486	Hypothetical protein
10	Moatr06Yg0188300.1	6	21142335	21143228	Retrovirus-related Pol polyprotein from transposon TNT 1-94
11	Moatr06Yg0188400.1	6	21147573	21148841	Gag/pol protein
12	Moatr06Yg0188500.1	6	21180733	21181197	Six-hairpin glycosidases superfamily protein
13	Moatr06Yg0188600.1	6	21197091	21199157	Pentatricopeptide repeat-containing protein
14	Moatr06Yg0188700.1	6	21216982	21220170	Gag/pol protein
15	Moatr06Yg0188900.1	6	21249450	21253400	Gag/pol protein
16	Moatr06Yg0189100.1	6	21992543	21992830	Pentatricopeptide repeat-containing protein
17	Moatr06Yg0189400.1	6	22180848	22181216	Unknown
18	Moatr06Yg0189500.1	6	22445601	22449551	Gag/pol protein
19	Moatr06Yg0189700.1	6	22513154	22514168	Retrovirus-related Pol polyprotein from transposon TNT 1-94
20	Moatr06Yg0189800.1	6	22534082	22537446	Gag/pol protein
21	Moatr06Yg0189900.1	6	22612052	22612237	Hypothetical protein
22	Moatr06Yg0190000.1	6	22703355	22703891	Pentatricopeptide repeat-containing protein
23	Moatr06Yg0190100.1	6	22885447	22888541	Hypothetical protein
24	Moatr06Yg0190400.1	6	23056150	23057183	Hypothetical protein
25	Moatr06Yg0190500.1	6	23147019	23147468	Unknown
26	Moatr06Yg0190600.1	6	23249603	23250449	Six-hairpin glycosidases superfamily protein
27	Moatr06Yg0190700.1	6	23362204	23363139	Six-hairpin glycosidases superfamily protein
28	Moatr06Yg0190800.2	6	23365039	23370743	Hypothetical protein
29	Moatr06Yg0190900.1	6	23500685	23501687	Hypothetical protein
30	Moatr06Yg0191000.1	6	23607196	23608176	Gag/pol protein
31	Moatr06Yg0191100.1		23653722	23654929	Pentatricopeptide repeat-containing protein
32	Moatr06Yg0191500.1		23769314	23770195	Unknown
33	Moatr06Yg0191600.1		23800110	23803207	Hypothetical protein
34	Moatr06Yg0191700.1		23991298	23991983	Pentatricopeptide repeat-containing protein
35	Moatr06Yg0191900.1		24064723	24064974	Hypothetical protein
36	Moatr06Yg0192300.1		24213629	24216055	Hypothetical protein
37	Moatr06Yg0192400.1		24264445	24265425	Unknown
38	Moatr06Yg0192600.1		24321548	24324599	Hypothetical protein
39	Moatr06Yg0192700.1		24486159	24486656	Hypothetical protein
40	Moatr06Yg0192800.1		24491511	24491960	Unknown
41	Moatr06Yg0192900.1		24569464	24570444	Unknown
42	Moatr06Yg0193100.1		24637205	24639016	Hypothetical protein

Table S16. Gene expression of sex-associated candidate genes specifically located in SDR-Y.

NO	Gene ID	T1[1]-NumReads		T2[2]-NumReads		T3[3]-NumReads	
		G225M[4]	G227F[5]	G225M	G227F	G225M	G227F
1	Moatr06Yg0184600.3	33.98	4.61	17.71	6.28	33.21	3.37
2	Moatr06Yg0184700.1	97.20	1.01	63.71	1.02	85.92	0.33
3	Moatr06Yg0184800.1	0.00	0	0.00	0.00	0.00	2.00
4	Moatr06Yg0184900.1	0.00	0	0.00	0.00	0.00	0.00
5	Moatr06Yg0187500.1	104.59	143.2	158.31	95.12	139.07	106.32
6	Moatr06Yg0187600.1	0.00	0	0.00	0.00	0.00	0.00
7	Moatr06Yg0187800.1	0.00	0	0.00	0.00	0.00	0.00
8	Moatr06Yg0188000.1	880.05	5.98	839.37	1.27	931.41	0.55
9	Moatr06Yg0188100.1	0.00	0	0.00	0.00	0.00	0.00
10	Moatr06Yg0188300.1	0.00	1.2	0.00	0.00	0.00	0.00
11	Moatr06Yg0188400.1	0.00	0	0.00	0.00	0.00	0.00
12	Moatr06Yg0188500.1	0.00	0	0.00	0.00	0.00	0.00
13	Moatr06Yg0188600.1	12.16	0	14.29	0.00	15.70	0.00
14	Moatr06Yg0188700.1	0.00	0	0.00	0.00	0.00	0.00
15	Moatr06Yg0188900.1	0.67	0	0.00	0.00	0.00	0.00
16	Moatr06Yg0189100.1	0.00	0	0.00	0.00	0.00	0.00
17	Moatr06Yg0189400.1	0.00	0	0.00	0.00	0.00	0.00
18	Moatr06Yg0189500.1	0.00	0.67	0.00	0.00	0.00	0.00
19	Moatr06Yg0189700.1	0.00	0.67	0.00	0.33	0.00	0.00
20	Moatr06Yg0189800.1	0.00	0	0.00	0.00	0.00	0.00
21	Moatr06Yg0189900.1	0.00	0	0.00	0.00	0.00	0.00
22	Moatr06Yg0190000.1	0.00	0	0.00	0.00	0.00	0.00
23	Moatr06Yg0190100.1	9.71	0.71	12.12	0.00	17.28	0.00
24	Moatr06Yg0190400.1	284.29	143.93	336.89	206.84	435.49	260.49
25	Moatr06Yg0190500.1	0.00	0	0.00	0.00	0.00	0.00
26	Moatr06Yg0190600.1	14.08	0	15.54	0.00	14.03	0.33
27	Moatr06Yg0190700.1	1.68	0	1.56	0.00	7.09	0.00
28	Moatr06Yg0190800.2	0.55	2.76	3.67	2.32	5.34	0.91
29	Moatr06Yg0190900.1	8.14	0	16.30	0.00	14.25	4.30
30	Moatr06Yg0191000.1	0.00	0	0.00	0.00	0.00	0.00
31	Moatr06Yg0191100.1	0.00	0	0.00	0.00	0.00	0.00
32	Moatr06Yg0191500.1	0.00	0	0.00	0.00	0.00	0.00
33	Moatr06Yg0191600.1	95.17	0	91.16	0.00	98.17	0.00
34	Moatr06Yg0191700.1	0.00	0	0.00	0.00	0.00	0.00
35	Moatr06Yg0191900.1	0.00	0	0.00	0.00	0.00	0.00
36	Moatr06Yg0192300.1	0.00	0	0.00	0.00	0.33	0.00
37	Moatr06Yg0192400.1	0.00	0	0.00	0.00	0.00	0.00
38	Moatr06Yg0192600.1	289.87	0	242.85	0.00	263.92	1.35
39	Moatr06Yg0192700.1	0.00	0	0.00	0.00	0.00	0.00
40	Moatr06Yg0192800.1	0.00	0	0.00	0.00	0.00	0.00
41	Moatr06Yg0192900.1	0.00	0	0.00	0.00	0.00	0.00
42	Moatr06Yg0193100.1	163.14	3.81	136.21	2.48	164.29	3.82

* [1] T1, stage of dormant bud sprouting; [2] T2, stage of early axillary bud; [3] T3, stage of late axillary bud; [4] G225M, Male individual; [5] G227F, Female individual.

Table S17. Identification of 30 functional genes located in GWAS significant region.

NO.	Genes located in candidate region	Chr.	Gene start	Gene end	SNP Number (-logP > 8)	Large-effect SNP		Gene annotation	*Arabidopsis* Ortholog
						Number	Type		
1	*EVM0019349.1*	6	21,261,862	21,263,449	1	0	/	Zinc finger protein AtZAT12	AT5G59820.1
2	*EVM0023019.1*	6	21,270,783	21,275,911	0	0	/	Copper/zinc superoxide dismutase 2	AT2G28190.1
3	*EVM0002384.1*	6	21,282,198	21,294,401	3	0	/	Hypothetical protein	AT1G54200.1
4	*EVM0001188.3*	6	21,295,180	21,300,190	1	0	/	Hypothetical protein	AT2G31600.3
5	*EVM0020947.1*	6	21,311,695	21,316,814	16	0	/	Flowering locus T protein	AT1G65480.1
6	*EVM0012351.2*	6	21,322,907	21,330,832	11	2	Non_synonymous; Splice_site_region	FASCIATA 1, Chromatin assembly factor group B	AT1G65470.2
7	*EVM0013876.1*	6	21,332,733	21,337,512	14	0	/	Suppressor of SA insensitive 2	AT2G43710.1
8	*EVM0006448.1*	6	21,337,977	21,339,095	4	1	Non_synonymous	Hypothetical protein	AT5G16250.1
9	*EVM0015954.1*	6	21,339,204	21,341,711	2	0	/	Ubiquitin-like protein ATG12	AT1G54210.1
10	*EVM0021739.1*	6	21,342,965	21,351,145	5	0	/	Early flowering protein 9	AT5G16260.1
11	*EVM0024092.1*	6	21,351,974	21,352,573	0	0	/	Hypothetical protein	AT5G03660.2
12	*EVM0018167.1*	6	21,360,607	21,362,272	0	0	/	GDSL-motif lipase 2	AT1G53940.2
13	*EVM0018384.1*	6	21,363,473	21,364,012	0	0	/	Hypothetical protein	AT5G41140.1
14	*EVM0008535.1*	6	21,365,979	21,371,137	1	0	/	Alkenal reductase	AT5G16970.1
15	*EVM0016434.1*	6	21,372,859	21,375,237	1	1	Non_synonymous	BAK1-interacting receptor-like kinase 1	AT5G48380.1
16	*EVM0022340.3*	6	21,376,222	21,384,905	9	2	Non_synonymous	Leucine zipper-EF-hand-containing transmembrane protein 2	AT1G65540.3
17	*EVM0014817.1*	6	21,386,652	21,395,529	4	0	/	Valine-tolerant 1	AT5G16290.1
18	*EVM0002637.2*	6	21,395,824	21,399,289	1	1	Non_synonymous	Repressor of silencing 3	AT5G58130.1
19	*EVM0011376.1*	6	21,403,506	21,413,840	35	10	Non_synonymous	Sister chromatid cohesion 1 protein	AT5G16270.1
20	*EVM0001895.1*	6	21,415,052	21,422,515	58	13	Non_synonymous; Splice_site_region	Beta-glucosidase 42	AT5G36890.1
21	*EVM0012542.1*	6	21,428,213	21,430,557	9	4	Non_synonymous	CYP79B2, cytochrome P450 79b2	AT4G39950.1
22	*EVM0025292.1*	6	21,450,368	21,452,676	8	4	Non_synonymous	CYP79A2, cytochrome p450 79a2	AT5G05260.2
23	*EVM0014931.1*	6	21,463,300	21,471,836	36	11	Non_synonymous; Stop_lost	Inositol-polyphosphate 5-phosphatase 12	AT2G43900.1
24	*EVM0017991.1*	6	21,485,988	21,491,342	21	5	Non_synonymous; Splice_site_region	Beta-hexosaminidase	AT1G65590.1
25	*EVM0000249.1*	6	21,491,461	21,494,387	5	1	Non_synonymous	Glycosyl hydrolase	AT1G65610.1
26	*EVM0022449.1*	6	21,495,411	21,503,963	23	3	Non_synonymous	Hypothetical protein	AT5G16300.1
27	*EVM0020853.1*	6	21,503,988	21,507,781	12	1	Non_synonymous	Protease Do-like 10	AT5G36950.1
28	*EVM0017089.1*	6	21,511,419	21,514,324	2	1	Non_synonymous	Hypothetical protein	AT1G21080.2
29	*EVM0010255.1*	6	21,516,950	21,520,568	11	0	/	LOB domain-containing protein	AT5G63090.3
30	*EVM0000383.1*	6	21,529,226	21,532,908	34	11	Non_synonymous; Stop_lost	Multiple organellar RNA editing factor 1	AT4G20020.2

Table S18. Gene expression of 30 functional genes located in GWAS significant region.

Gene ID	T1[1]-FPKM				T2[2]-FPKM			
	G225M[3]	G227F[4]	log_2(M/F)	*P*-value	G225M	G227F	log_2(M/F)	*P*-value
EVM0019349.1	4.07	0.83	2.30	2.81E-06	2.49	3.90	-0.65	/
EVM0023019.1	63.72	83.61	-0.39	/	58.96	40.74	0.53	/
EVM0002384.1	9.08	9.03	0.01	/	7.70	9.36	-0.28	/
EVM0001188.3	22.82	20.24	0.17	/	14.42	15.48	-0.10	/
EVM0020947.1	0.39	1.16	-1.56	3.84E-03	0.71	0.86	-0.28	/
EVM0012351.2	12.21	15.62	-0.36	/	5.18	2.55	1.02	3.95E-04
EVM0013876.1	37.33	20.40	0.87	3.20E-12	17.79	17.72	0.01	/
EVM0006448.1	60.90	183.73	-1.59	2.26E-08	162.46	95.33	0.77	4.44E-04
EVM0015954.1	12.99	14.76	-0.18	/	10.06	10.84	-0.11	/
EVM0021739.1	25.26	22.86	0.14	/	17.02	21.46	-0.33	/
EVM0024092.1	12.33	0.00	6.95	2.13E-46	0.00	0.00	0.00	/
EVM0018167.1	190.17	0.00	10.89	3.90E-53	0.00	0.00	0.00	/
EVM0018384.1	28.01	0.00	8.13	5.51E-57	0.00	0.00	0.00	/
EVM0008535.1	47.58	13.44	1.82	5.18E-26	19.44	18.00	0.11	/
EVM0016434.1	11.90	13.92	-0.23	/	21.29	20.07	0.09	/
EVM0022340.3	20.26	19.93	0.02	/	20.08	20.90	-0.06	/
EVM0014817.1	31.17	41.74	-0.42	/	13.74	11.68	0.23	/
EVM0002637.2	13.51	12.54	0.11	/	12.01	11.23	0.10	/
EVM0011376.1	27.14	28.56	-0.07	/	28.09	27.98	0.01	/
EVM0001895.1	26.78	38.61	-0.53	/	59.82	132.48	-1.15	3.67E-14
EVM0012542.1	0.35	1.45	-2.03	5.03E-03	0.02	31.35	-10.90	5.93E-131
EVM0025292.1	0.05	0.40	-2.94	9.31E-03	0.18	0.23	-0.32	/
EVM0014931.1	16.52	14.52	0.19	/	15.57	18.66	-0.26	/
EVM0017991.1	22.80	42.86	-0.91	9.29E-08	10.14	16.97	-0.74	1.20E-03
EVM0000249.1	0.04	0.27	-2.86	2.85E-02	0.20	0.14	0.47	/
EVM0022449.1	33.32	31.37	0.09	/	32.36	38.61	-0.25	/
EVM0020853.1	15.02	20.86	-0.47	/	10.84	8.46	0.36	/
EVM0017089.1	0.06	0.08	-0.40	/	0.00	0.00	0.00	/
EVM0010255.1	32.11	8.79	1.87	9.94E-06	2.06	2.22	-0.11	/
EVM0000383.1	48.28	61.91	-0.36	/	45.69	44.62	0.03	/

* [1] T1, stage of dormant bud sprouting; [2] T2, stage of axillary bud; [3] G225M, Male individual; [4] G227F, Female individual.

Additional file 1: Datas

Data 1. Summary of all accessions sequenced in this study (see separate files).

Data 2. Admix proportion for each hybrid (see separate files**).**

Data 3 Analysis of Patterson's D between different areas (see separate files).

Data 4 SNP analysis associated with sex (-log10(*P*) > 8) (see separate files).

Data 5 Statistics of the heterozygosity level of the 558 sex associated SNPs (-log10(*P*) > 8) (see separate files).

Data 6 Functional annotation of significant expanded gene families (*P* < 0.05) (see separate files**).**

Data 7 Functional annotation of significant contraction gene families (*P* < 0.05) (see separate files**).**
